# Surveillance for highly pathogenic avian influenza A (H5N1) in a raptor rehabilitation center—2022

**DOI:** 10.1371/journal.pone.0299330

**Published:** 2024-04-29

**Authors:** Victoria Hall, Carol Cardona, Kristelle Mendoza, Mia Torchetti, Kristina Lantz, Irene Bueno, Dana Franzen-Klein

**Affiliations:** 1 The Raptor Center, College of Veterinary Medicine, University of Minnesota, Saint Paul, Minnesota, United States of America; 2 College of Veterinary Medicine, University of Minnesota, Saint Paul, Minnesota, United States of America; 3 United States Department of Agriculture Animal and Plant Health Inspection Services, National Veterinary Services Laboratories, Veterinary Services, Ames, Iowa, United States of America; 4 Bristol Veterinary School, University of Bristol, Langford, Bristol, England; Universidad Cooperativa de Colombia, COLOMBIA

## Abstract

An ongoing, severe outbreak of highly pathogenic avian influenza virus (HPAI) A H5N1 clade 2.3.4.4b has been circulating in wild and domestic bird populations throughout the world, reaching North America in 2021. This HPAI outbreak has exhibited unique characteristics when compared to previous outbreaks. The global distribution of disease, prolonged duration, extensive number of species and individual wild birds affected, and the large impact on the global poultry industry have all exceeded historical impacts of previous outbreaks in North America. In this study, we describe the results of HPAI surveillance conducted at The Raptor Center, a wildlife rehabilitation hospital at University of Minnesota (Saint Paul, MN, U.S.A.), from March 28th–December 31, 2022. All wild raptors admitted to the facility were tested for avian influenza viruses using polymerase chain reaction (PCR) testing. All non-negative samples were submitted to the United States Department of Agriculture (USDA) Animal and Plant Health Inspection Service (APHIS) National Veterinary Services Laboratories for confirmatory HPAI testing and genetic sequencing. During the study period, 996 individual birds representing 20 different species were tested for avian influenza, and 213 birds were confirmed HPAI positive. Highly pathogenic avian influenza surveillance conducted at The Raptor Center contributed 75% of the HPAI positive raptor detections within the state of Minnesota, located within the Mississippi flyway, significantly augmenting state wildlife surveillance efforts. The viral genotypes observed in birds sampled at The Raptor Center were representative of what was seen in wild bird surveillance within the Mississippi flyway during the same time frame. Wildlife rehabilitation centers provide an opportune situation to augment disease surveillance at the human, wildlife and domestic animal interface during ongoing infectious disease outbreaks.

## Introduction

Highly pathogenic avian influenza (HPAI) A H5N1 clade 2.3.4.4b virus caused numerous outbreaks in domestic poultry and wild birds globally in 2022 [1–3]. The virus was introduced to North America from Europe in December 2021 [4], and since then, has spread throughout the continent.

Within the United States (U.S.), Minnesota is known for its role in poultry agriculture, being the nation’s top turkey producer, while also producing broilers, eggs, and upland gamebirds [5]. Minnesota is also located in the Mississippi flyway, which is a major migratory corridor for wild birds [6]. It is estimated that 40% of North America’s waterfowl and shorebirds traverse the Mississippi flyway each year [6]. The geographic overlap of poultry production and over 230 wild bird species makes Minnesota a potential hotspot for the introduction of influenza viruses, including HPAI, via wild birds during migratory staging and nesting. In the 2022 H5N1 epizootic, the first poultry cases confirmed as H5 2.3.4.4b lineage in the state were on March 25, and the first detected wild bird cases were on March 30, 2022 [4,7]. By the end of 2022, 81 commercial and 29 backyard poultry flocks (4,220,141 poultry) had been infected with HPAI within the state of Minnesota, with substantial associated economic impacts [8]. During that same timeframe, 554 individual wild birds representing 37 species were confirmed H5N1 positive within the state of Minnesota [2]. Throughout the U.S. in 2022, over 53 million poultry and close to 6,000 wild birds, representing over 130 different species, were confirmed H5N1 positive [2,8].

Quantification of the effect of such an outbreak in domestic poultry is relatively straightforward due to the availability of data from regulatory agencies, poultry companies, and backyard producers. However, estimating the number of wild birds affected by this same outbreak proves much more challenging despite increased surveillance by multiple agencies and collaboration in reporting from the public. The number and variety of wild birds impacted by HPAI are likely substantially underestimated given the conditions in the natural environment, remote areas where birds may have succumbed to disease, limited wild bird testing capacity, and varied clinical presentations among the species impacted. Thus, there are potentially many wild birds unaccounted for when estimating the toll of the outbreak.

One of the ways to improve the surveillance of wildlife diseases, such as HPAI, is the use of wildlife rehabilitation centers. The Raptor Center (TRC), at the University of Minnesota (Saint Paul, MN, raptor.umn.edu) admits approximately 1,000 raptors every year. Raptors are a group of avian species that generally have severe clinical outcomes when infected with HPAI viruses [9–12]. As of July 1, 2023, raptor deaths during the 2023 HPAI epizootic have been reported in 27 species and 48 states [2]. To better understand the nature of the outbreak and its effect on raptors, TRC conducted active surveillance of all birds admitted to the center from the beginning of the outbreak in Minnesota.

This study describes HPAI surveillance conducted in a raptor rehabilitation center in Minnesota, U.S. from March 28th–December 31, 2022. The results of this study provide helpful insight into the epidemiology of the disease in 20 raptor species, which in turn, helps us to better understand the use of wild bird surveillance in wildlife rehabilitation centers, especially amid disease outbreaks.

## Materials and methods

The Raptor Center receives an average of 1,000 ill, injured, and orphaned raptors (eagles, hawks, owls, falcons, vultures, osprey) per year. All wildlife rehabilitation activities are conducted under state and federal permits. Disease testing and medical treatments described in this publication did not require institutional animal care and use committee approval as it was part of routine clinical patient assessment and care. The majority of birds admitted during the study period were recovered from the state of Minnesota. A smaller proportion of individuals were admitted from the neighboring states of Wisconsin, North Dakota and Iowa. Once a bird was identified as injured or sick, the public, a partner organization, or TRC volunteers captured and transported the bird to TRC for evaluation. During transport, the birds were kept in individual containment, most commonly cardboard boxes or pet carriers. Both live and deceased birds were accepted for evaluation.

Upon arrival to TRC, birds were maintained in individual housing in a bio-secure triage and quarantine area separate from the main facility. On the day of admission, a full medical examination was performed by a trained raptor veterinarian and medical treatment was initiated as appropriate for the individual bird. Strict biosecurity, quarantine, and disinfection protocols were utilized to limit the risk of disease transmission between individual patients, and to prevent movement of the disease outside of the facility. Biosecurity protocols for all patients included: initial admission in a bio-secure building, a quarantine period in a cohort group with dedicated equipment for each cohort, multiple avian influenza (AI) polymerase chain reaction (PCR) tests including one test at admission and repeated testing during and at the end of quarantine, use of personal protective equipment (PPE) for all staff entering the triage and quarantine area (consisting of hooded full body waterproof suits, goggles, respirators, and gloves), disinfection protocols using an accelerated hydrogen peroxide disinfectant (Rescue^®^, Virox technologies Inc., Ontario Canada), and further enhanced disinfection and testing specifically around HPAI suspect or confirmed positive birds.

From March 28 through December 31, 2022, all birds that presented to TRC were screened for influenza A virus with reverse-transcriptase polymerase chain reaction (rRT-PCR) testing targeting conserved region(s) of the matrix gene following standard methods [13,14]. Duplicate swab samples were collected from the oropharyngeal and cloacal cavities of each bird as part of their diagnostic clinical evaluation. Oropharyngeal swabs only were collected on some individuals weighing under 200g. Swabs were placed in brain-heart infusion (BHI) media and kept at 4°C if testing was to be completed within 24 hours of sample collection, or at -18°C if testing was to be completed greater than 24 hours after sample collection. Duplicate tests were run at the University of Minnesota Veterinary Diagnostic Lab (Saint Paul, MN) and at The Raptor Center Wild Bird Diagnostic Lab (Saint Paul, MN). All non-negative samples were forwarded to the United States Department of Agriculture (USDA) Animal and Plant Health Inspection Service (APHIS) National Veterinary Services Laboratories (NVSL, APHIS USDA, Ames, Iowa) for subtyping and sequencing.

Testing at NVSL included H5 clade 2.3.4.4 and N1 rapid subtyping [15]. Influenza A viruses were sequenced directly from samples as previously described [4] and randomized axelerated maximum likelihood (RaxML) was used to generate phylogenetic trees. Tables of single nucleotide polymorphisms (SNPs) were created using the vSNP pipeline (github.com/USDA-VS/vSNP). Laboratory results from raptors tested from TRC were compiled with results of wild bird surveillance in the Mississippi flyway, also tested at NVSL.

The Raptor Center surveillance results from 2022 were descriptively compared with TRC surveillance results from the last HPAI outbreak in the state of Minnesota in 2015 (unpublished data). The 2015 surveillance efforts were focused on a passive surveillance system of two groups: 1) opportunistic testing of raptors entering rehab when resources were available and 2) targeted testing, when possible, of clinical cases where HPAI was a possible diagnosis, including any individual bird or group of birds that were inconsistent with normal patterns of admission, or any individual bird with clinical signs consistent with neurological and/or respiratory impairment not attributable to trauma or another well-recognized disease or syndrome (West Nile Virus, lead intoxication, aspergillosis, etc.). Samples were screened for influenza as previously described with rRT-PCR by the University of Minnesota Veterinary Diagnostic Laboratory.

## Results

From March 28 –December 31, 2022, TRC conducted complete, active HPAI surveillance of all raptor admissions, which included 996 raptors representing 20 species. Of the 996 birds, 848 presented alive and 148 were dead on arrival. Nine-hundred and six individuals were from the state of Minnesota (originating from 73 of the 87 counties in the state), 82 from Wisconsin (originating from 16 of the 72 counties in the state), six from North Dakota (originating from 2 of the 53 counties in the state), and one from Iowa (Table 1).

**Table 1:** Summary of highly pathogenic avian influenza (HPAI) polymerase chain reaction (PCR) test results by state and county during the study period of March 28 –December 31, 2022.

Minnesota					
County	Number of Birds Tested	Number of HPAI Positive Birds	County	Number of Birds Tested	Number of HPAI Positive Birds
Anoka*	57	22	Marshall	2	0
Becker	2	0	Martin	4	1
Beltrami	1	0	McLeod	15	3
Benton	6	0	Meeker	8	2
Big Stone	2	1	Mille Lacs	4	0
Blue Earth	9	1	Morrison	4	3
Brown	4	0	Mower	3	0
Carlton	2	0	Murray	1	0
Carver*	18	6	Nicollet	5	1
Cass	2	0	Nobles	3	0
Chippewa	1	0	Olmsted	16	2
Chisago*	13	6	Otter Tail	6	1
Clay	4	0	Pennington	1	0
Clearwater	2	0	Pine	11	2
Cook	1	0	Polk	4	0
Corcoran	1	0	Ramsey*	83	22
Cottonwood	4	0	Redwood	3	0
Crow Wing	2	0	Renville	4	0
Dakota*	65	15	Rice	7	2
Dodge	2	0	Roseau	1	1
Douglas	2	0	Scott*	30	5
Faribault	4	1	Sherburne	30	5
Fillmore	8	0	Sibley	2	0
Freeborn	7	0	St. Louis	24	1
Goodhue	21	6	Stearns	13	2
Hennepin*	204	51	Steele	6	0
Houston	5	1	Swift	2	0
Hubbard	2	0	Todd	4	1
Isanti	8	2	Unknown	6	0
Itasca	4	0	Wabasha	12	1
Jackson	1	0	Wadena	2	1
Kanabec	4	1	Waseca	4	0
Kandiyohi	6	1	Washington*	63	21
Lac qui Parle	1	0	Watonwan	2	0
Le Sueur	7	2	Winona	13	1
Lincoln	1	0	Wright	22	5
Lyon	2	0	Yellow Medicine	1	0
			**Totals**:	906	199
**Wisconsin**					
**County**	**Number of Birds Tested**	**Number of HPAI Positive Birds**	**County**	**Number of Birds Tested**	**Number of HPAI Positive Birds**
Bayfield	2	0	Pepin	3	0
Buffalo	4	0	Pierce	14	3
Burnett	1	0	Polk	14	3
Chippewa	2	0	Sawyer	2	1
Dunn	3	1	St. Croix	24	4
Eau Claire	3	0	Trempealeau	4	2
Jackson	1	0	Unknown	1	0
La Crosse	2	0	Washburn	1	0
Monroe	1	0	**Totals**:	82	14
**North Dakota**			**Iowa**		
**County**	**Number of Birds Tested**	**Number of HPAI Positive Birds**	**County**	**Number of Birds Tested**	**Number of HPAI Positive Birds**
Cass	4	0	Cherokee	1	0
Grand Forks	3	0	**Totals**:	1	0
**Totals**:	7	0			

Counties within the seven county Minneapolis/Saint Paul metropolitan area are indicated with an asterisk (*).

Of the 996 birds, 783 (78.6%) tested negative for HPAI. All birds that tested negative on their admission PCR sample tested negative on repeat PCR tests, confirming that biosecurity methods were sufficient to prevent disease transmission within the wildlife hospital.

Of the 996 total birds, 213 (21.4%) were confirmed HPAI positive. Of the positive testing birds, 93% were recovered from Minnesota (n = 199) and 7% from Wisconsin (n = 14) (Table 1). Positive birds represented 12 species, with the top three species being great horned owls (*Bubo virginianus*, n = 92*)*, bald eagles (*Haliaeetus leucocephalus*, n = 48*)*, and red-tailed hawks (*Buteo jamaicensis*, n = 39) (Table 2). An additional eight species tested negative during surveillance (Table 2).

**Table 2:** Summary of highly pathogenic avian influenza (HPAI) polymerase chain reaction (PCR) test results by species during the study period of March 28 –December 31, 2022.

Species	Number of Birds Tested	Number of HPAI Positive Birds	% Positive
American kestrel (*Falco sparverius*)	16	1	6%
Bald eagle (*Haliaeetus leucocephalus*)	165	48	29%
Barred owl (*Strix varia*)	81	7	9%
Broad-winged hawk (*Buteo platypterus*)	71	1	1%
Cooper’s hawk (*Accipiter cooperii*)	118	6	5%
Eastern screech-owl (*Megascops asio*)	15	0	0%
Great horned owl (*Bubo virginianus*)	196	92	47%
Long-eared owl (*Asio otus*)	4	0	0%
Merlin (*Falco columbarius*)	32	0	0%
Northern goshawk (*Accipiter gentilis*)	3	0	0%
Northern saw-whet owl (*Aegolius acadicus*)	6	0	0%
Northern harrier (*Circus hudsonius*)	3	1	33%
Osprey (*Pandion haliaetus*)	15	0	0%
Peregrine falcon (*Falco peregrinus*)	24	8	33%
Red-shouldered hawk (*Buteo lineatus*)	16	5	31%
Red-tailed hawk (*Buteo jamaicensis*)	190	39	21%
Rough-legged hawk (*Buteo lagopus*)	3	1	33%
Short-eared owl (*Asio flammeus*)	1	0	0%
Sharp-shinned hawk (*Accipiter striatus*)	15	0	0%
Turkey vulture (*Cathartes aura*)	23	4	17%

Of the 213 HPAI positive birds, 133 (62%) presented alive and 80 (38%) presented dead on arrival. Of the birds alive at admission, 83% had some clinical sign on evaluation or epidemiologic link suggestive of HPAI exposure, 16% had no suggestive indicators, and 1.5% died before a full evaluation could be done. One adult female great horned owl that was confirmed HPAI positive made a full clinical recovery, stopped shedding avian influenza virus on repeat PCR testing, and was released back to the wild. All other HPAI PCR positive birds either died or were humanely euthanized due to severity of disease.

**Fig 1:**
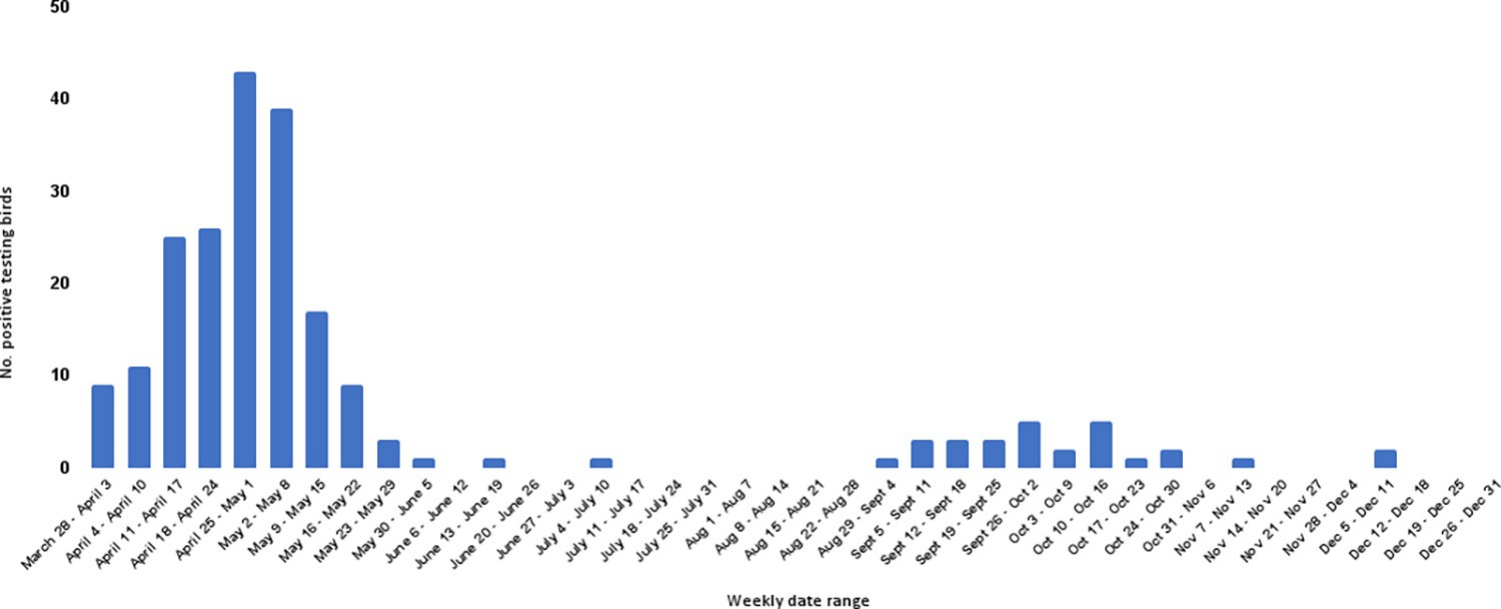
Highly pathogenic avian influenza (HPAI) polymerase chain reaction (PCR) positive birds admitted to TRC by week—March 28–December 31, 2022.

**Fig 2:**
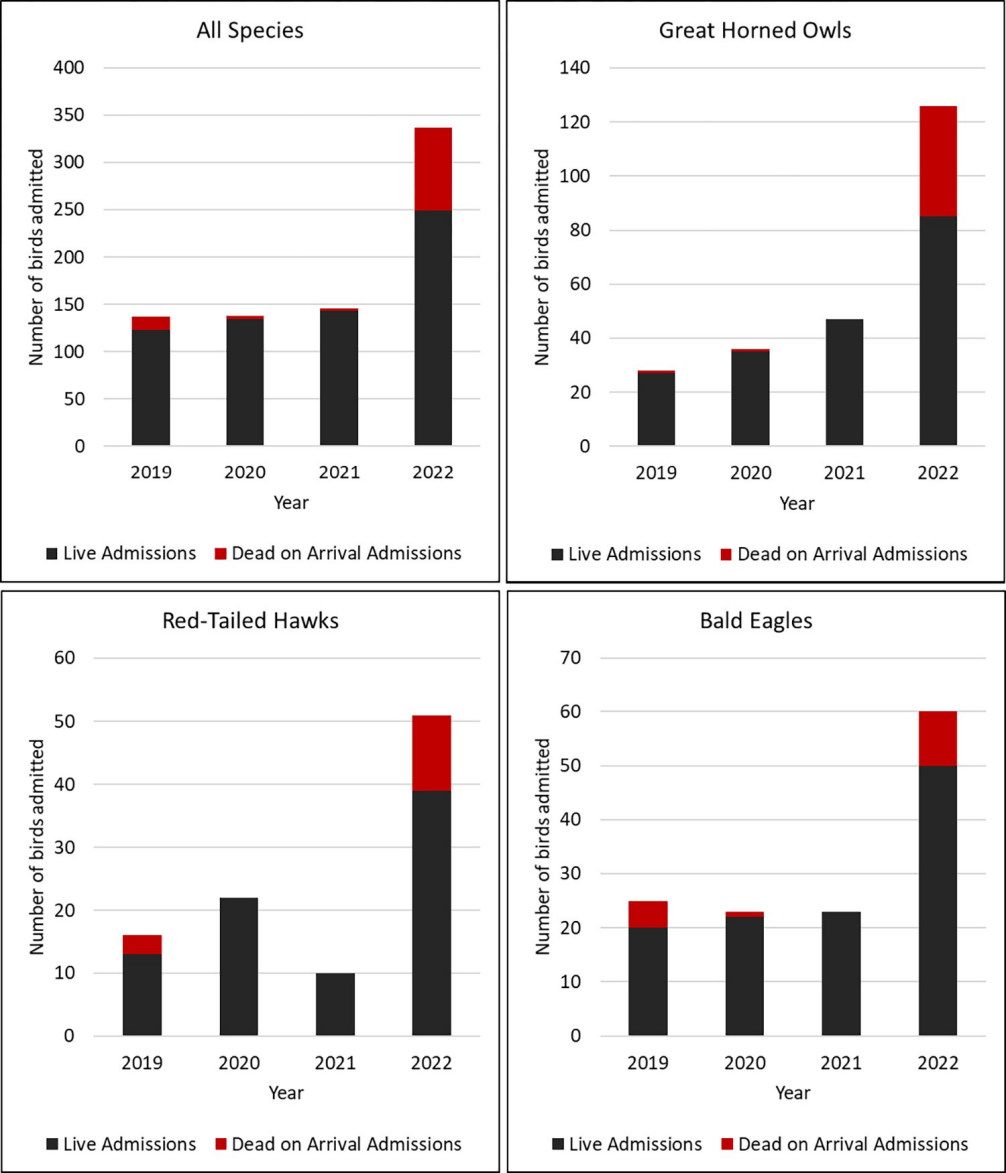
Four year summary, during the interval of March 28–May 31, of wild bird admissions to The Raptor Center subdivided by species and live vs dead on arrival admissions.

The most substantial surge of positive cases occurred from March 28 –May 31, 2022, with a second smaller increase in case count noted from August 29 –October 30, 2022 (Fig 1). During the spring surge from March 28 –May 31, 2022, TRC admitted more birds during this time frame than is typical for the season when compared to the previous three years (Fig 2). The Raptor Center saw a nearly two fold increase in the number of live patient admissions and close to a 30 fold increase in the number of birds that presented dead on arrival during the spring of 2022 (Fig 2).

**Fig 3:**
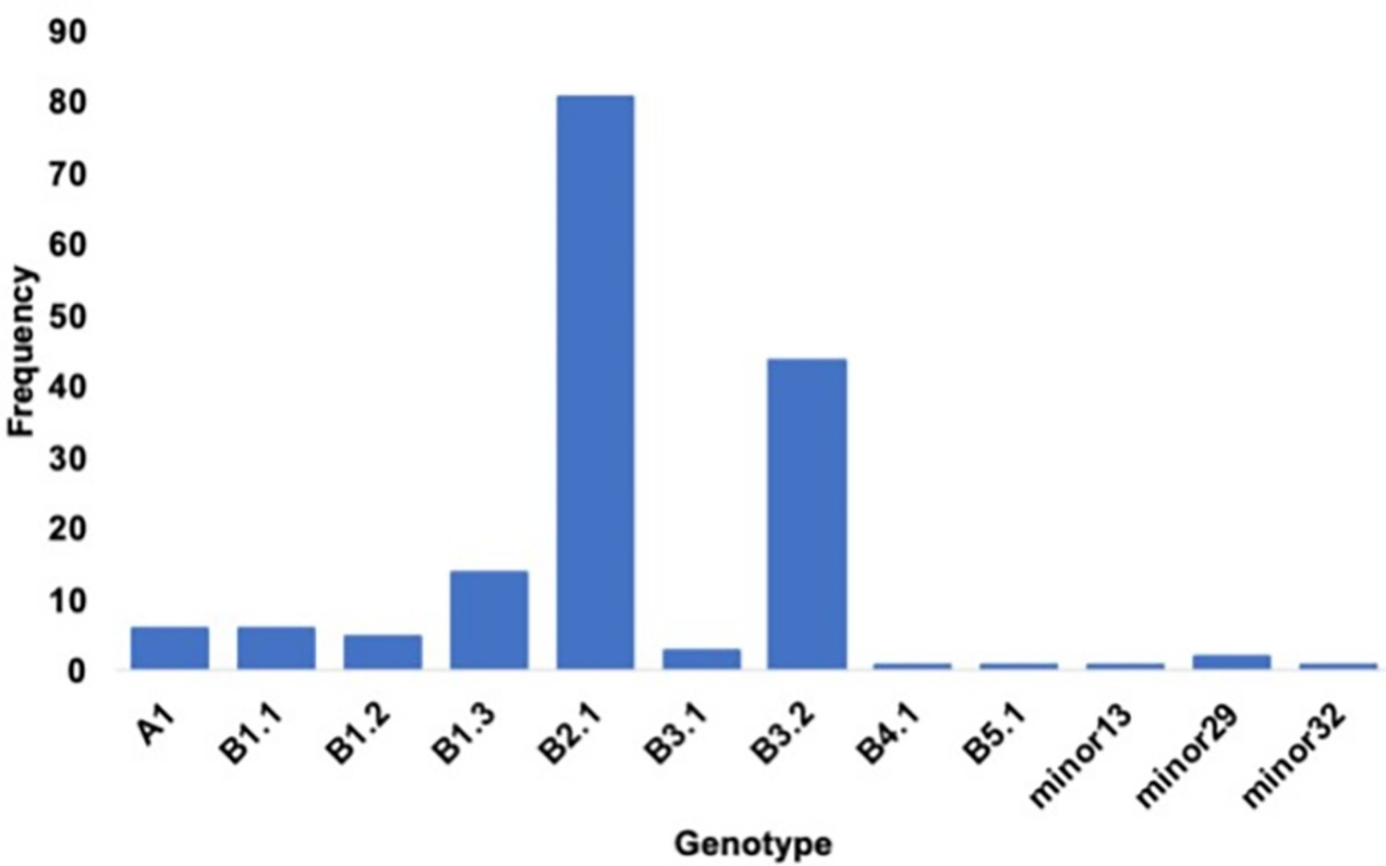
Highly pathogenic avian influenza (HPAI) viral genotypes detected in wild bird admissions to The Raptor Center—March 28–December 31, 2022.

**Fig 4:**
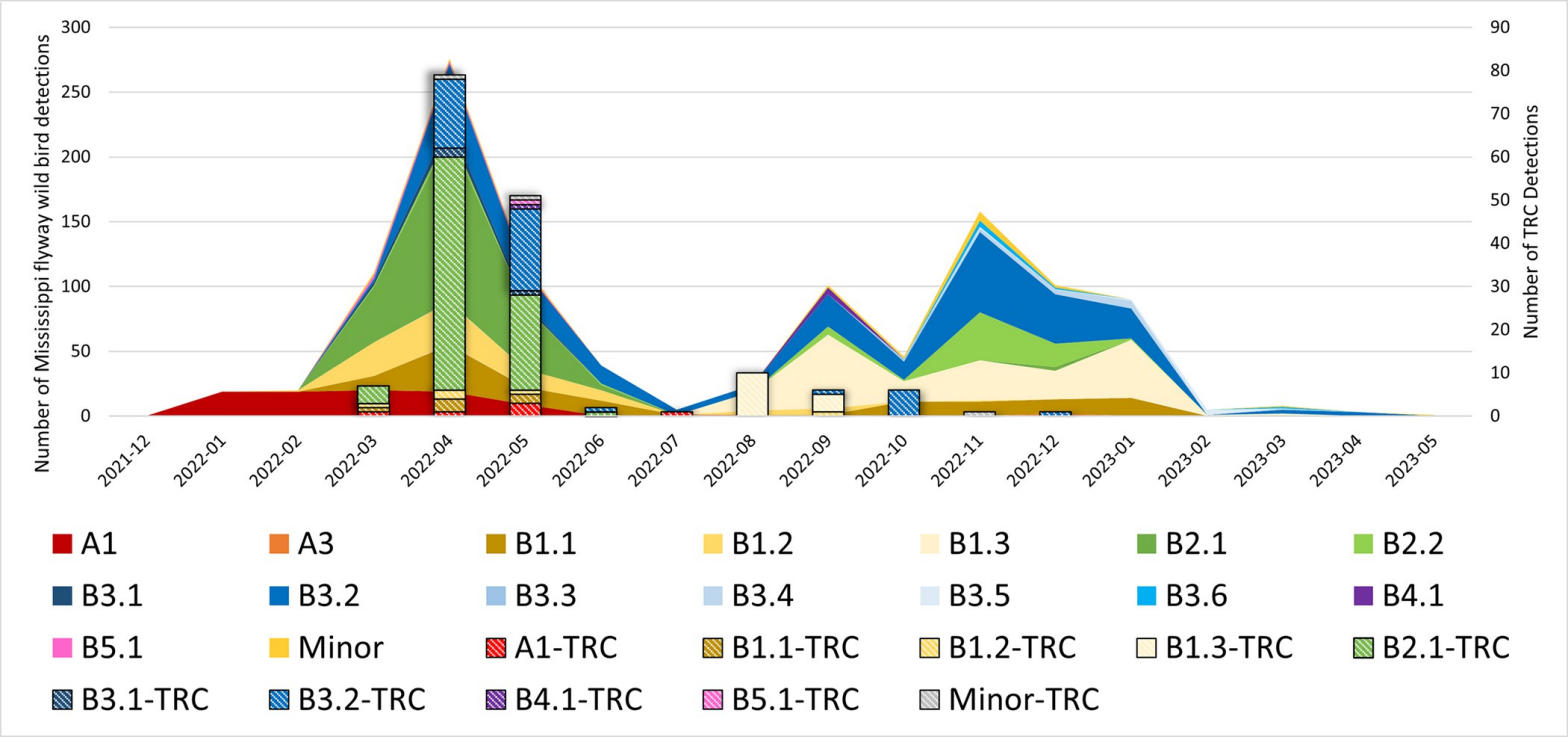
Mississippi flyway HPAI wild bird detections by genotype (area graph) and TRC HPAI detections by genotype (bar graph)—December 2021–May 2023. The HPAI genotypes detected by NVSL in wild bird sampling conducted within the Mississippi flyway is depicted in the area graph, while the HPAI genotypes detected through TRC surveillance is depicted in the bar graph. Scales have been adjusted for each to illustrate proportional data.

Full genome sequence data was generated directly from 165 samples representing nine genotypes and three episodic strains (Fig 3). No markers of mammalian adaptation were observed in any of the sequences when utilizing the PB2 gene and markers E627K, D701N, T217A, and 591K for monitoring. Viral genotype data from TRC birds was mapped over the sampling period and compared to general wild bird surveillance results from the Mississippi flyway to evaluate trends in types (Fig 4). Similar peaks in detection and similar proportions of various genotypes were noted between wild bird surveillance in the Mississippi flyway and in TRC surveillance results from March through December 2022 (Fig 4 and S1 Table). Representative sequences have been uploaded to NCBI and Genbank and are indicated where available (S1 Table).

In contrast to the 2022 PCR data in this study, from January 1—December 31, 2015, TRC tested 268 wild raptors that were admitted to TRC for care, representing 11 raptor species, for avian influenza viruses via PCR. No samples were positive for HPAI during the 2015 HPAI outbreak in Minnesota.

## Discussion

The outbreak of HPAI H5N1 clade 2.3.4.4b virus that arrived to North America late in 2021 has exhibited markedly different epidemiological characteristics and impacts on wild birds than previous global and North American HPAI outbreaks [16]. With positive poultry detections in 47 states and wild bird detections in 49 states, it is more geographically widespread than any previous outbreak [8]. There is more involvement of backyard poultry flocks than seen historically, with over 550 positive flock detections thus far within the U.S., suggesting a greater wild bird transmission component to the outbreak [8]. In wild birds, we are seeing greater numbers of individuals and species impacted than previously, as well as marked spill over into a variety of mammalian species, which has never been observed before in North America [8].

Disease surveillance of wild birds upon admission to wildlife rehabilitation facilities is a valuable opportunity to characterize disease and gain information on disease transmission within local wild bird populations. The value of conducting surveillance at this interface is demonstrated when TRC’s HPAI surveillance data from the 2015 and 2022 outbreaks are compared. During TRC’s 2015 surveillance, no positive testing raptors were detected. Similarly, state led surveillance in 2015 included 104 morbidity and mortality samples from wild birds and found only a single HPAI positive raptor, a Cooper’s hawk (*A*. *cooperii*) [17]. In contrast, in 2022 substantially more disease transmission was noted among wild bird populations in the region, and TRC detected 199 HPAI positive raptors from Minnesota, and a total of 213 positive birds including birds recovered from Wisconsin. The state of Minnesota, in total, reported 251 detections of HPAI in raptor species through all 2022 surveillance systems, meaning that TRC surveillance efforts contributed to over 75% of raptor detections in the state, greatly augmenting state surveillance data. While TRC surveillance efforts in 2015 focused on testing clinically ill birds, we know from 2022 testing that the majority of HPAI virus-infected raptors that presented to rehab for care were symptomatic. Though 2015 surveillance was not as robust as it was in 2022, it would have likely detected at least some positive individuals if the virus was circulating in wild raptors at significant levels.

The three most common HPAI positive raptor species admitted to TRC were great horned owls, bald eagles, and red-tailed hawks. These are the three most common species historically admitted to TRC each year. However, admission rates for these species during the spring of 2022 were dramatically increased compared to previous years. This increase in cases could be attributed to an actual marked increase in ill birds due to the HPAI outbreak. Admission rates may have also been influenced by substantial media attention surrounding the outbreak that may have made members of the public more likely to identify sick birds and contact TRC for assistance.

The diet of great horned owls and bald eagles is a potential route of disease exposure for these raptors, as they commonly eat natural reservoir species for avian influenza, such as waterfowl. Great horned owls are opportunistic hunters, eating a wide range of prey items including waterfowl [18,19]. Bald eagles also have a varied diet including fish, mammals and avian prey items; they regularly consume waterfowl species and will scavenge on carrion that could include birds that have died from HPAI [20,21]. Conversely, the diet of red-tailed hawks is primarily composed of small mammals, small birds, and occasionally pheasants and quail [22]. Red-tailed hawks, as well as some other species that tested HPAI positive during TRC’s surveillance such as the American kestrel and broad-winged hawk, do not routinely consume HPAI reservoir species, warranting further investigation into additional routes of disease exposure.

The predominant genotypes of the HPAI viruses from raptors admitted to TRC were the Eurasian (EA) A1 (genotype of the first introduction), and EA/North American (AM) reassortants B2.1 and B3.2. These genotypes were well represented across the top three species, and were also the predominant genotypes in wild birds in the region at the time of sample collection. Genotype B2.1 is a two gene reassortant (AM PB2, NP) that dominated cases in the Mississippi flyway in wild birds and poultry during the spring of 2022 and detections decreased dramatically by June of 2022. The most predominant genotype nationwide has been B3.2 which has AM PB2, PB1, NP, NS genes. This genotype has had a steady presence across all four flyways in the U.S. since the spring of 2022 and expanded to become the predominant genotype in the fall of 2022.

Proportionally similar trends were noted in viral genotype prevalence and positive bird numbers between TRC surveillance results and general surveillance results from wild birds in the Mississippi flyway between March and December 2022. When HPAI is circulating amongst wild bird populations, it is expected for the disease to increase in prevalence during spring and fall migrations as wild birds gather in large groups which facilitates disease transmission [4,7,15,16]. This is the trend that was observed in TRC’s wild bird surveillance in 2022, as well as wild bird surveillance conducted throughout the United States [2]. March is often the start of the spring wild bird migration into the state of Minnesota, with November marking the end of fall migration [23]. After the end of October in 2022, TRC positive bird detections dramatically decreased after migratory birds had left the state. National surveillance numbers continued to rise through late fall 2022 as those migratory bird populations continued their journey south, where the disease continued to circulate and be detected on surveillance sampling.

One limitation of using wildlife rehabilitation centers for insight into disease dynamics within the larger wild bird population, is that the population entering care and ultimately included in data collection are animals that are 1) identified by a person as in need of help, 2) debilitated enough to be successfully captured, and 3) able to be transported to the rehabilitation facility [24–26]. This creates bias in the population that is admitted and sampled, as injured or sick birds that are located near larger populations of people are more likely to be found, and birds that are found close to a wildlife rehabilitation center have a greater chance of being transported to the center for care. This was noted in TRC’s surveillance data, as 59% of birds admitted from the state of Minnesota during the surveillance period originated in the seven county Minneapolis/Saint Paul metropolitan area, which is home to approximately 60% of Minnesota’s state population, and is the geographic location of The Raptor Center’s hospital. While most admissions came from this population dense area, TRC did ultimately admit birds from 84% of the counties within the state of Minnesota, and TRC data comprised the majority of positive raptor detections within the state.

## Conclusions

Wildlife rehabilitation facilities provide opportunities to augment greater disease surveillance efforts at the human, wild bird, domestic bird interface. As the main task of these facilities is to accept ill and injured birds, they are more likely to receive infected birds during disease outbreaks. Additionally, because wildlife hospitals are already handling the birds to provide medical care, this creates convenient opportunities for sample and data collection. The viral genotype trends observed via surveillance at TRC were comparable to the viral genotypes observed in wild bird surveillance within the same geographic region, which further supports that data collected from wildlife sampled upon admission into a wildlife rehabilitation hospital can contribute valuable information when assessing ongoing disease outbreaks within local wild bird populations.

## Supporting information

S1 Table National Veterinary Services Laboratory (NVSL) identification number and genotype for all viral genotypes depicted in Fig 4; NCBI and Genbank referral numbers are provided where available. (DOCX)
