# Supplementary material for: Surveillance for highly pathogenic avian influenza A (H5N1) in a raptor rehabilitation center—2022
Source: PLoS One. 2024 Apr 29;19(4):e0299330. doi: 10.1371/journal.pone.0299330 (PMC11057742; doi:10.1371/journal.pone.0299330)
Supplement: S1 Table — (DOCX) [file pone.0299330.s001.docx]

| **NVSL ID** | **Genotype** | **Referral number (SRR NCBI; EPI GISAID)** |
| --- | --- | --- |
| 22-004941-003 | A1 | SRR24839738 |
| 22-005153-004 | A1 | SRR24839066 |
| 22-005154-004 | A1 | SRR24839059 |
| 22-005907-002 | A1 | SRR24839711 |
| 22-011586-001 | B2.1 | SRR24843186 |
| 22-011586-002 | B2.1 | SRR24843185 |
| 22-011586-003 | B2.1 | SRR24842924 |
| 22-011586-004 | B2.1 | SRR24842922 |
| 22-011678-001 | A1 | SRR24842916 |
| 22-011695-001 | Minor01 | SRR24842915 |
| 22-004809-001 | A1 | SRR24839740 |
| 22-004809-002 | A1 | SRR24839739 |
| 22-005907-002 | A1 | SRR24839711 |
| 22-005910-002 | A1 | SRR24839393 |
| 22-005910-004 | A1 | SRR24839373 |
| 22-006328-004 | A1 | SRR24839381 |
| 22-006328-011 | A1 | SRR24839380 |
| 22-006328-015 | A1 | SRR24839379 |
| 22-006328-019 | A1 | SRR24839378 |
| 22-006328-022 | A1 | SRR24839377 |
| 22-006328-032 | A1 | SRR24839376 |
| 22-006742-003 | A1 | SRR24839352 |
| 22-006970-001 | A1 | SRR24839337 |
| 22-007184-001 | A1 | SRR24839654 |
| 22-007184-001 | A1 | SRR24839654 |
| 22-007187-001 | B1.2 | SRR24839653 |
| 22-007265-001 | A1 | SRR24839674 |
| 22-007469-001 | B1.2 | SRR24839647 |
| 22-007482-001 | B2.1 | SRR24839547 |
| 22-007551-001 | B2.1 | SRR24839542 |
| 22-007679-001 | B2.1 | SRR24839538 |
| 22-007679-003 | B2.1 | SRR24839536 |
| 22-007679-005 | B2.1 | SRR24839644 |
| 22-007679-006 | B2.1 | SRR24839643 |
| 22-007679-007 | B2.1 | SRR24839642 |
| 22-007679-008 | B2.1 | SRR24839533 |
| 22-007679-009 | B2.1 | SRR24839532 |
| 22-007679-010 | B2.1 | SRR24839531 |
| 22-007691-001 | A1 | SRR24839530 |
| 22-007697-001 | B1.2 | SRR24839520 |
| 22-007698-001 | B3.1 | SRR24839519 |
| 22-007698-002 | B2.1 | SRR24839518 |

| 22-007698-003 | B5.1 | SRR24839645 |
| --- | --- | --- |
| 22-007700-001 | B2.1 | SRR24839624 |
| 22-007700-003 | B2.1 | SRR24839623 |
| 22-007700-004 | B2.1 | SRR24839622 |
| 22-007948-001 | B1.2 | SRR24839324 |
| 22-007764-001 | A1 | SRR24839621 |
| 22-007765-001 | A1 | SRR24839641 |
| 22-007766-003 | B2.1 | SRR24839640 |
| 22-008376-001 | B2.1 | SRR24839504 |
| 22-008376-002 | A1 | SRR24839503 |
| 22-008377-002 | A1 | SRR24839502 |
| 22-008377-004 | A1 | Unpublished data |
| 22-008377-005 | A1 | SRR24839501 |
| 22-008378-001 | A1 | SRR24839499 |
| 22-008379-001 | B2.1 | SRR24839498 |
| 22-008568-001 | B1.2 | SRR24839599 |
| 22-008506-001 | B1.2 | SRR24839611 |
| 22-008506-002 | B1.2 | SRR24839610 |
| 22-008506-003 | B1.2 | SRR24839609 |
| 22-008506-004 | B1.2 | SRR24839608 |
| 22-008506-005 | B1.2 | SRR24839607 |
| 22-008506-006 | B1.2 | SRR24839606 |
| 22-008506-007 | B1.2 | SRR24839604 |
| 22-008506-008 | B1.2 | SRR24839602 |
| 22-008506-009 | B1.2 | SRR24839601 |
| 22-008719-001 | B2.1 | SRR24839283 |
| 22-008719-002 | B3.1 | SRR24839590 |
| 22-008719-003 | B2.1 | SRR24839486 |
| 22-008719-004 | B3.1 | Unpublished data |
| 22-008995-001 | B1.1 | SRR24839256 |
| 22-008995-002 | B1.1 | SRR24839255 |
| 22-009205-005 | B1.2 | SRR24839570 |
| 22-009205-010 | A1 | SRR24839569 |
| 22-009207-002 | A1 | SRR24839568 |
| 22-009207-003 | A1 | SRR24839567 |
| 22-009207-012 | B1.2 | SRR24839566 |
| 22-009209-007 | A1 | SRR24839565 |
| 22-009209-011 | A1 | SRR24839564 |
| 22-009209-012 | A1 | SRR24839563 |
| 22-009381-001 | B2.1 | SRR24839168 |
| 22-009383-001 | B2.1 | SRR24839167 |
| 22-009404-001 | B2.1 | SRR24839481 |
| 22-009404-002 | A1 | SRR24839479 |
| 22-009407-001 | B1.1 | SRR24839477 |
| 22-009408-001 | A1 | SRR24839458 |
| 22-009411-001 | Minor01 | SRR24839456 |
| 22-009477-001 | B3.2 | SRR24839471 |

| 22-009361-003 | B1.2 | SRR24839562 |
| --- | --- | --- |
| 22-009361-004 | B1.2 | SRR24839186 |
| 22-009361-005 | B1.2 | SRR24839183 |
| 22-009361-006 | B1.2 | SRR24839182 |
| 22-009361-007 | B1.2 | SRR24839181 |
| 22-009361-008 | B1.2 | SRR24839556 |
| 22-009361-009 | B1.2 | SRR24839178 |
| 22-009361-011 | B1.1 | SRR24839177 |
| 22-009361-012 | B1.2 | SRR24839176 |
| 22-009361-014 | B1.2 | SRR24839175 |
| 22-009361-015 | B1.2 | SRR24839174 |
| 22-009361-017 | B1.1 | SRR24839173 |
| 22-009668-001 | B3.2 | SRR24839204 |
| 22-009684-001 | B1.1 | SRR24839202 |
| 22-009930-002 | B2.1 | SRR24839445 |
| 22-010027-001 | B2.1 | SRR24839407 |
| 22-010027-002 | B2.1 | SRR24839406 |
| 22-010027-003 | B2.1 | SRR24839405 |
| 22-010027-004 | B1.1 | SRR24839428 |
| 22-010027-005 | B2.1 | SRR24839404 |
| 22-010027-006 | B1.2 | SRR24839403 |
| 22-010027-007 | A1 | SRR24839401 |
| 22-010028-001 | B1.1 | SRR24839400 |
| 22-010035-001 | B2.1 | SRR24839396 |
| 22-010035-003 | B2.1 | SRR24839395 |
| 22-010129-001 | A1 | SRR24839113 |
| 22-010206-001 | B2.1 | SRR24839684 |
| 22-010232-001 | B2.1 | SRR24839682 |
| 22-010236-001 | A1 | SRR24839678 |
| 22-010305-001 | B2.1 | SRR24839696 |
| 22-010305-002 | B2.2 | Unpublished data |
| 22-010354-001 | B3.2 | SRR24839762 |
| 22-010355-002 | B3.2 | SRR24839761 |
| 22-010620-001 | B2.1 | SRR24843256 |
| 22-010620-002 | B2.1 | SRR24843242 |
| 22-010666-001 | B2.1 | SRR24843000 |
| 22-010667-001 | A1 | SRR24843313 |
| 22-010713-002 | B2.1 | SRR24843288 |
| 22-010713-003 | B2.1 | Unpublished data |
| 22-010713-004 | B2.1 | SRR24842845 |
| 22-010715-001 | B1.1 | SRR24843266 |
| 22-010922-001 | B1.2 | SRR24842819 |
| 22-010922-002 | B1.2 | Unpublished data |
| 22-010924-001 | B2.1 | SRR24843097 |
| 22-010925-001 | B1.2 | Unpublished data |
| 22-010927-001 | B2.1 | SRR24843096 |
| 22-011023-001 | B2.1 | SRR24843422 |

| 22-011026-001 | Minor01 | SRR24843423 |
| --- | --- | --- |
| 22-011028-001 | B2.1 | SRR24843424 |
| 22-011029-001 | B3.2 | SRR24843240 |
| 22-011029-002 | B1.1 | SRR24843241 |
| 22-011148-007 | B2.1 | Unpublished data |
| 22-011224-001 | B2.1 | SRR24843395 |
| 22-011225-001 | B3.2 | SRR24843394 |
| 22-011226-001 | B2.1 | SRR24843393 |
| 22-011227-001 | B2.1 | SRR24843415 |
| 22-011228-001 | B3.2 | SRR24843413 |
| 22-011229-001 | B2.1 | SRR24843412 |
| 22-011230-001 | B2.1 | SRR24843411 |
| 22-011231-001 | B2.1 | SRR24843410 |
| 22-011232-001 | B3.1 | SRR24843409 |
| 22-011237-001 | B2.1 | SRR24843408 |
| 22-011238-001 | B2.1 | SRR24843407 |
| 22-011239-001 | A1 | SRR24843406 |
| 22-011240-001 | B1.1 | SRR24843405 |
| 22-011250-001 | B1.1 | SRR24843404 |
| 22-011251-001 | B1.1 | SRR24843401 |
| 22-011252-001 | B2.1 | SRR24843400 |
| 22-011298-001 | B2.1 | SRR24843214 |
| 22-011346-001 | B2.1 | SRR24843225 |
| 22-011350-001 | B2.1 | SRR24843221 |
| 22-011360-001 | B1.1 | SRR24843402 |
| 22-011404-001 | B2.1 | SRR24843216 |
| 22-011517-001 | B1.2 | SRR24843202 |
| 22-011517-002 | B1.2 | SRR24843201 |
| 22-011517-003 | B1.2 | SRR24843200 |
| 22-011517-004 | B1.1 | SRR24843199 |
| 22-011517-006 | B1.2 | SRR24843198 |
| 22-011517-007 | B1.2 | SRR24843197 |
| 22-011517-008 | B1.1 | SRR24843388 |
| 22-011517-009 | B1.2 | SRR24843386 |
| 22-011517-010 | A1 | SRR24843385 |
| 22-011517-011 | B1.2 | SRR24843384 |
| 22-011586-001 | B2.1 | SRR24843186 |
| 22-011586-002 | B2.1 | SRR24843185 |
| 22-011586-003 | B2.1 | SRR24842924 |
| 22-011586-004 | B2.1 | SRR24842922 |
| 22-011101-001 | A1 | SRR24843068 |
| 22-011101-003 | A1 | SRR24843067 |
| 22-011101-005 | A1 | Unpublished data |
| 22-011101-006 | A1 | Unpublished data |
| 22-011101-007 | A1 | SRR24843066 |
| 22-011101-008 | A1 | Unpublished data |
| 22-011101-009 | A1 | Unpublished data |

| 22-011814-001 | B2.1 | SRR24843166 |
| --- | --- | --- |
| 22-011817-001 | B3.2 | SRR24843021 |
| 22-011818-001 | B2.1 | SRR24843019 |
| 22-011819-001 | B2.1 | SRR24843018 |
| 22-011820-001 | B2.1 | SRR24843040 |
| 22-011821-001 | B2.1 | Unpublished data |
| 22-011824-001 | B1.1 | SRR24843039 |
| 22-011825-001 | B1.2 | SRR24843037 |
| 22-011871-001 | B3.2 | SRR24843030 |
| 22-011871-002 | B2.1 | SRR24843029 |
| 22-011871-004 | B3.2 | SRR24843028 |
| 22-011871-006 | B2.1 | SRR24843026 |
| 22-011871-008 | B2.1 | SRR24843025 |
| 22-011871-009 | B2.1 | SRR24843024 |
| 22-011871-010 | B2.1 | SRR24843023 |
| 22-011871-011 | B2.1 | SRR24843022 |
| 22-011871-012 | B2.1 | SRR24843020 |
| 22-011871-013 | B2.1 | SRR24843348 |
| 22-011871-014 | B2.1 | SRR24843347 |
| 22-011948-001 | A1 | SRR24843355 |
| 22-011948-002 | A1 | SRR24843354 |
| 22-011949-001 | B2.2 | SRR24843353 |
| 22-011949-002 | B2.2 | SRR24843352 |
| 22-012000-002 | B2.1 | SRR24843351 |
| 22-012000-003 | B3.2 | SRR24843349 |
| 22-012000-004 | B2.1 | SRR24843332 |
| 22-012003-001 | B3.2 | SRR24843331 |
| 22-012003-002 | B2.1 | SRR24843330 |
| 22-012007-001 | B2.1 | SRR24843328 |
| 22-012022-002 | B2.1 | SRR24843326 |
| 22-012026-001 | B2.1 | SRR24843344 |
| 22-012028-001 | B2.1 | SRR24843343 |
| 22-012029-001 | B3.2 | SRR24843342 |
| 22-012030-001 | B2.1 | SRR24843341 |
| 22-012031-001 | B2.1 | SRR24843340 |
| 22-012032-001 | B3.2 | SRR24843339 |
| 22-012056-001 | B1.2 | SRR24842911 |
| 22-012056-002 | B2.1 | SRR24842910 |
| 22-012056-004 | B1.1 | SRR24842909 |
| 22-012056-005 | B1.2 | SRR24842907 |
| 22-012056-006 | A1 | SRR24842906 |
| 22-012056-007 | A1 | Unpublished data |
| 22-012056-009 | B1.1 | SRR24842905 |
| 22-012093-001 | B3.2 | SRR24842895 |
| 22-012305-001 | B3.2 | SRR24843161 |
| 22-012338-001 | B5.1 | SRR24843148 |
| 22-012338-002 | B5.1 | SRR24843146 |

| 22-012338-004 | B2.1 | SRR24843144 |
| --- | --- | --- |
| 22-012338-005 | B2.1 | SRR24843143 |
| 22-012338-008 | B2.1 | SRR24843140 |
| 22-012338-015 | B2.1 | SRR24843009 |
| 22-012338-023 | B2.1 | SRR24843004 |
| 22-012338-024 | B5.1 | SRR24843003 |
| 22-012338-025 | B1.1 | SRR24843001 |
| 22-012414-001 | B2.1 | SRR24842992 |
| 22-012416-001 | B2.1 | SRR24842991 |
| 22-012668-001 | B2.1 | SRR24843307 |
| 22-012668-002 | B3.2 | SRR24843306 |
| 22-012668-003 | B2.1 | SRR24843305 |
| 22-012668-005 | B2.1 | SRR24843304 |
| 22-012668-006 | B2.1 | SRR24843302 |
| 22-012668-007 | B2.1 | SRR24843301 |
| 22-012668-009 | B2.1 | SRR24843300 |
| 22-012668-010 | B2.1 | SRR24843299 |
| 22-012668-011 | B2.1 | SRR24843298 |
| 22-012668-012 | B2.1 | Unpublished data |
| 22-012668-013 | B3.2 | SRR24843297 |
| 22-012668-014 | B2.1 | SRR24843296 |
| 22-012668-015 | B2.1 | SRR24843295 |
| 22-012668-016 | B3.1 | SRR24843294 |
| 22-012670-001 | B2.1 | SRR24842862 |
| 22-012670-002 | B2.1 | SRR24842860 |
| 22-012671-001 | B2.2 | SRR24842859 |
| 22-012759-001 | B2.1 | Unpublished data |
| 22-012759-002 | B2.1 | SRR24842877 |
| 22-012759-003 | B2.1 | SRR24842876 |
| 22-012760-003 | B2.1 | SRR24842875 |
| 22-012771-001 | B1.1 | SRR24842871 |
| 22-012771-002 | B2.1 | SRR24842870 |
| 22-012771-003 | B1.1 | SRR24842869 |
| 22-012771-004 | B1.1 | SRR24842868 |
| 22-012772-001 | B2.1 | SRR24842867 |
| 22-012773-003 | B3.2 | SRR24842866 |
| 22-012775-001 | B2.1 | SRR24842865 |
| 22-012777-001 | B2.1 | Unpublished data |
| 22-012777-002 | B3.1 | Unpublished data |
| 22-012854-001 | B1.2 | SRR24842863 |
| 22-012854-002 | B1.2 | SRR24843120 |
| 22-012854-003 | B1.1 | SRR24842858 |
| 22-012854-006 | B1.2 | SRR24842857 |
| 22-012897-001 | B1.2 | SRR24842851 |
| 22-012931-001 | B2.1 | SRR24843132 |
| 22-012931-004 | B3.2 | SRR24843131 |
| 22-012931-005 | B2.1 | SRR24843129 |

| 22-012931-006 | B2.1 | SRR24843128 |
| --- | --- | --- |
| 22-012931-007 | B2.1 | SRR24843127 |
| 22-012931-008 | B2.1 | Unpublished data |
| 22-012931-009 | B3.2 | SRR24843126 |
| 22-012931-010 | B2.1 | SRR24843125 |
| 22-012969-001 | B1.1 | SRR24843112 |
| 22-013050-001 | B2.1 | SRR24842977 |
| 22-013058-001 | B1.2 | SRR24842969 |
| 22-013128-001 | B2.1 | SRR24842959 |
| 22-013128-002 | B1.1 | SRR24842958 |
| 22-013128-003 | B2.1 | SRR24842957 |
| 22-013128-004 | B2.1 | SRR24842956 |
| 22-013128-005 | B2.1 | Unpublished data |
| 22-013128-007 | B1.1 | SRR24842955 |
| 22-013257-001 | B2.1 | Unpublished data |
| 22-013257-002 | B2.1 | Unpublished data |
| 22-013261-002 | B2.1 | Unpublished data |
| 22-013261-003 | B3.2 | Unpublished data |
| 22-013261-004 | B3.2 | Unpublished data |
| 22-013261-005 | B3.2 | Unpublished data |
| 22-013262-001 | A1 | Unpublished data |
| 22-013262-002 | B3.2 | Unpublished data |
| 22-013267-001 | B2.1 | Unpublished data |
| 22-013269-001 | A1 | Unpublished data |
| 22-013271-001 | B1.1 | Unpublished data |
| 22-013272-001 | B2.1 | Unpublished data |
| 22-013273-001 | B1.1 | Unpublished data |
| 22-013275-001 | B1.1 | Unpublished data |
| 22-013276-001 | B2.1 | Unpublished data |
| 22-013277-001 | B1.1 | Unpublished data |
| 22-013277-002 | B1.1 | Unpublished data |
| 22-013278-001 | B2.1 | Unpublished data |
| 22-013394-001 | B3.1 | Unpublished data |
| 22-013394-002 | B2.1 | Unpublished data |
| 22-013395-002 | B3.2 | Unpublished data |
| 22-013395-003 | B2.1 | Unpublished data |
| 22-013395-007 | B3.2 | Unpublished data |
| 22-013395-008 | B3.2 | Unpublished data |
| 22-013395-009 | B3.2 | Unpublished data |
| 22-013395-010 | B2.1 | Unpublished data |
| 22-013395-011 | B1.1 | Unpublished data |
| 22-013395-012 | B2.1 | Unpublished data |
| 22-013395-013 | B2.1 | Unpublished data |
| 22-013396-001 | B2.1 | Unpublished data |
| 22-013397-001 | B2.1 | Unpublished data |
| 22-013397-002 | B3.2 | Unpublished data |
| 22-013424-001 | B1.1 | Unpublished data |

| 22-013424-002 | B1.1 | Unpublished data |
| --- | --- | --- |
| 22-013424-003 | B1.1 | Unpublished data |
| 22-013425-001 | B2.1 | Unpublished data |
| 22-013425-002 | B2.1 | Unpublished data |
| 22-013425-003 | B2.1 | Unpublished data |
| 22-013426-001 | B2.1 | Unpublished data |
| 22-013428-001 | B3.1 | Unpublished data |
| 22-013429-001 | B2.1 | Unpublished data |
| 22-013431-001 | B2.1 | Unpublished data |
| 22-013432-001 | B2.1 | Unpublished data |
| 22-013433-001 | B2.1 | Unpublished data |
| 22-013434-001 | B2.1 | Unpublished data |
| 22-013435-001 | A1 | Unpublished data |
| 22-013436-001 | B3.2 | Unpublished data |
| 22-013517-001 | B1.1 | Unpublished data |
| 22-013517-002 | B1.2 | Unpublished data |
| 22-013517-003 | B1.2 | Unpublished data |
| 22-013517-007 | B1.1 | Unpublished data |
| 22-013517-010 | A1 | Unpublished data |
| 22-013517-011 | B1.2 | Unpublished data |
| 22-013517-012 | B1.1 | Unpublished data |
| 22-013544-001 | B3.1 | Unpublished data |
| 22-013544-002 | B2.1 | Unpublished data |
| 22-012383-001 | B1.2 | Unpublished data |
| 22-012387-001 | B2.1 | Unpublished data |
| 22-012388-001 | B1.1 | Unpublished data |
| 22-012390-001 | B1.1 | Unpublished data |
| 22-012391-001 | B2.1 | Unpublished data |
| 22-012392-001 | B2.1 | Unpublished data |
| 22-012396-001 | B1.2 | Unpublished data |
| 22-013558-001 | B2.1 | Unpublished data |
| 22-013642-001 | B1.2 | Unpublished data |
| 22-013642-002 | B1.1 | Unpublished data |
| 22-013662-001 | B2.1 | Unpublished data |
| 22-013662-003 | B2.1 | Unpublished data |
| 22-013663-002 | A1 | Unpublished data |
| 22-013663-004 | B3.2 | Unpublished data |
| 22-013663-005 | B2.1 | Unpublished data |
| 22-013663-006 | B3.2 | Unpublished data |
| 22-013663-007 | B2.1 | Unpublished data |
| 22-013664-001 | minor13 | Unpublished data |
| 22-013666-001 | B2.1 | Unpublished data |
| 22-013667-001 | B3.2 | Unpublished data |
| 22-013669-001 | B2.1 | Unpublished data |
| 22-013672-001 | B2.1 | Unpublished data |
| 22-013751-001 | B2.1 | Unpublished data |
| 22-013751-002 | B3.2 | Unpublished data |

| 22-013751-003 | B1.2 | Unpublished data |
| --- | --- | --- |
| 22-013751-004 | B2.1 | Unpublished data |
| 22-013751-005 | B2.1 | Unpublished data |
| 22-013957-002 | B1.1 | Unpublished data |
| 22-013957-003 | B2.1 | Unpublished data |
| 22-013984-001 | B1.1 | Unpublished data |
| 22-013984-002 | B1.1 | Unpublished data |
| 22-014037-001 | B2.1 | Unpublished data |
| 22-014037-002 | B2.1 | Unpublished data |
| 22-014037-004 | B3.2 | Unpublished data |
| 22-014183-003 | B3.2 | Unpublished data |
| 22-014183-004 | B3.2 | Unpublished data |
| 22-014232-001 | B2.1 | Unpublished data |
| 22-014260-001 | B2.2 | Unpublished data |
| 22-014336-001 | Minor29 | Unpublished data |
| 22-014336-002 | B2.1 | Unpublished data |
| 22-014336-003 | B2.1 | Unpublished data |
| 22-014336-004 | B3.2 | Unpublished data |
| 22-014336-007 | B2.1 | Unpublished data |
| 22-014336-008 | B2.1 | Unpublished data |
| 22-014336-009 | B3.2 | Unpublished data |
| 22-014336-010 | B2.1 | Unpublished data |
| 22-014336-011 | B5.1 | Unpublished data |
| 22-014336-012 | B2.1 | Unpublished data |
| 22-014336-013 | B2.1 | Unpublished data |
| 22-014336-015 | B3.2 | Unpublished data |
| 22-014352-001 | B3.2 | Unpublished data |
| 22-014353-001 | B2.1 | Unpublished data |
| 22-014354-001 | B3.2 | Unpublished data |
| 22-014355-001 | B2.1 | Unpublished data |
| 22-014356-001 | B2.1 | Unpublished data |
| 22-014359-001 | B1.2 | Unpublished data |
| 22-014359-002 | B1.2 | Unpublished data |
| 22-014359-003 | B1.2 | Unpublished data |
| 22-014361-001 | B1.2 | Unpublished data |
| 22-014360-001 | B1.1 | Unpublished data |
| 22-014362-001 | B3.2 | Unpublished data |
| 22-014363-001 | B2.1 | Unpublished data |
| 22-014365-001 | B3.2 | Unpublished data |
| 22-014366-001 | B3.2 | Unpublished data |
| 22-014367-001 | B3.2 | Unpublished data |
| 22-014369-001 | B3.2 | Unpublished data |
| 22-014370-001 | B1.2 | Unpublished data |
| 22-014371-001 | B1.2 | Unpublished data |
| 22-014372-001 | B5.1 | Unpublished data |
| 22-014373-001 | B1.2 | Unpublished data |
| 22-014374-001 | B2.1 | Unpublished data |

| 22-014377-001 | B2.1 | Unpublished data |
| --- | --- | --- |
| 22-014378-001 | B1.1 | Unpublished data |
| 22-014378-002 | B1.1 | Unpublished data |
| 22-014393-001 | B1.1 | Unpublished data |
| 22-014394-001 | B2.1 | Unpublished data |
| 22-014404-001 | B3.2 | Unpublished data |
| 22-014405-001 | B3.2 | Unpublished data |
| 22-014416-001 | B3.2 | Unpublished data |
| 22-014468-001 | B2.1 | Unpublished data |
| 22-014469-003 | B2.1 | Unpublished data |
| 22-014510-001 | B2.1 | Unpublished data |
| 22-014513-001 | A1 | Unpublished data |
| 22-014517-001 | A1 | Unpublished data |
| 22-014518-001 | B2.1 | Unpublished data |
| 22-014519-001 | B2.1 | Unpublished data |
| 22-014520-001 | A1 | Unpublished data |
| 22-014521-001 | B3.2 | Unpublished data |
| 22-014521-002 | B3.2 | Unpublished data |
| 22-014522-001 | B3.1 | Unpublished data |
| 22-014524-001 | B2.1 | Unpublished data |
| 22-014525-001 | B3.2 | Unpublished data |
| 22-014526-001 | B2.1 | Unpublished data |
| 22-014528-001 | B2.1 | Unpublished data |
| 22-014529-001 | B3.2 | Unpublished data |
| 22-014649-001 | B2.1 | Unpublished data |
| 22-014658-004 | B3.2 | Unpublished data |
| 22-014658-005 | Minor29 | Unpublished data |
| 22-014658-007 | B3.2 | Unpublished data |
| 22-014899-001 | A1 | Unpublished data |
| 22-015051-001 | B2.1 | Unpublished data |
| 22-015095-001 | B3.2 | Unpublished data |
| 22-015095-002 | B1.1 | Unpublished data |
| 22-015197-001 | B2.1 | Unpublished data |
| 22-015197-002 | B3.2 | Unpublished data |
| 22-015310-001 | B2.1 | Unpublished data |
| 22-015311-001 | B2.1 | Unpublished data |
| 22-015312-001 | B3.2 | Unpublished data |
| 22-015314-001 | B3.2 | Unpublished data |
| 22-015317-001 | B2.1 | Unpublished data |
| 22-015318-001 | B2.1 | Unpublished data |
| 22-015319-001 | B2.1 | Unpublished data |
| 22-015320-001 | B2.1 | Unpublished data |
| 22-015321-001 | B2.1 | Unpublished data |
| 22-015322-001 | B2.1 | Unpublished data |
| 22-015323-001 | B2.1 | Unpublished data |
| 22-015458-001 | B3.2 | Unpublished data |
| 22-015545-001 | A1 | Unpublished data |

| 22-013673-001 | B3.2 | Unpublished data |
| --- | --- | --- |
| 22-014028-001 | B1.2 | Unpublished data |
| 22-015675-001 | A1 | Unpublished data |
| 22-015783-001 | B4.1 | Unpublished data |
| 22-015801-001 | B1.1 | Unpublished data |
| 22-011871-007 | B3.1 | Unpublished data |
| 22-014379-001 | B1.1 | Unpublished data |
| 22-015312-001 | B3.2 | Unpublished data |
| 22-016225-001 | B3.2 | Unpublished data |
| 22-016226-001 | B2.1 | Unpublished data |
| 22-016227-001 | B3.2 | Unpublished data |
| 22-016228-001 | B3.1 | Unpublished data |
| 22-016277-001 | B3.2 | Unpublished data |
| 22-016279-001 | B3.2 | Unpublished data |
| 22-016280-001 | B2.1 | Unpublished data |
| 22-016280-002 | B2.1 | Unpublished data |
| 22-016281-001 | B2.1 | Unpublished data |
| 22-016283-001 | B2.1 | Unpublished data |
| 22-016284-001 | B3.2 | Unpublished data |
| 22-016285-001 | B2.1 | Unpublished data |
| 22-016286-001 | B2.1 | Unpublished data |
| 22-016287-001 | B2.1 | Unpublished data |
| 22-016288-001 | B2.1 | Unpublished data |
| 22-016539-001 | B1.2 | Unpublished data |
| 22-016540-002 | B1.2 | Unpublished data |
| 22-016541-001 | B1.2 | Unpublished data |
| 22-016701-001 | B2.1 | Unpublished data |
| 22-016894-001 | B1.2 | Unpublished data |
| 22-017052-001 | B1.1 | Unpublished data |
| 22-017052-005 | B1.1 | Unpublished data |
| 22-017059-001 | B1.1 | Unpublished data |
| 22-017059-002 | B1.1 | Unpublished data |
| 22-017059-003 | B1.1 | Unpublished data |
| 22-017060-001 | B2.1 | Unpublished data |
| 22-017060-002 | B2.1 | Unpublished data |
| 22-017243-001 | B1.2 | Unpublished data |
| 22-017243-004 | B1.1 | Unpublished data |
| 22-017243-005 | B1.1 | Unpublished data |
| 22-017243-006 | B1.1 | Unpublished data |
| 22-017243-007 | B1.2 | Unpublished data |
| 22-017243-010 | B1.2 | Unpublished data |
| 22-017243-011 | B1.2 | Unpublished data |
| 22-017243-012 | B1.2 | Unpublished data |
| 22-017243-013 | B1.2 | Unpublished data |
| 22-017243-015 | B1.2 | Unpublished data |
| 22-017243-020 | B2.1 | Unpublished data |
| 22-017243-021 | B2.1 | Unpublished data |

| 22-017243-022 | B1.2 | Unpublished data |
| --- | --- | --- |
| 22-017243-023 | B2.1 | Unpublished data |
| 22-017243-024 | B2.1 | Unpublished data |
| 22-017550-001 | B1.2 | Unpublished data |
| 22-017717-001 | B1.2 | Unpublished data |
| 22-017717-002 | B1.2 | Unpublished data |
| 22-017717-003 | B1.1 | Unpublished data |
| 22-017717-004 | B1.1 | Unpublished data |
| 22-017717-005 | B1.2 | Unpublished data |
| 22-017769-001 | B3.2 | Unpublished data |
| 22-017940-001 | B3.2 | Unpublished data |
| 22-017940-003 | B3.2 | Unpublished data |
| 22-017940-004 | B3.2 | Unpublished data |
| 22-017940-005 | B3.2 | Unpublished data |
| 22-017940-006 | B2.1 | Unpublished data |
| 22-017940-007 | B3.2 | Unpublished data |
| 22-017940-008 | B3.2 | Unpublished data |
| 22-017940-009 | B3.2 | Unpublished data |
| 22-017940-010 | B3.2 | Unpublished data |
| 22-018184-001 | B1.2 | Unpublished data |
| 22-018423-001 | B3.2 | Unpublished data |
| 22-018423-002 | B3.2 | Unpublished data |
| 22-018423-004 | B3.2 | Unpublished data |
| 22-018423-005 | B3.2 | Unpublished data |
| 22-018552-001 | B3.2 | Unpublished data |
| 22-018806-001 | B1.1 | Unpublished data |
| 22-018806-002 | B1.2 | Unpublished data |
| 22-018806-003 | B1.2 | Unpublished data |
| 22-018806-004 | B1.2 | Unpublished data |
| 22-018806-005 | B1.2 | Unpublished data |
| 22-018986-002 | B1.1 | Unpublished data |
| 22-019074-001 | B3.2 | Unpublished data |
| 22-019204-001 | A1 | Unpublished data |
| 22-019205-001 | B1.1 | Unpublished data |
| 22-019205-002 | B1.1 | Unpublished data |
| 22-019205-005 | B1.1 | Unpublished data |
| 22-019205-006 | B1.1 | Unpublished data |
| 22-019205-007 | B2.1 | Unpublished data |
| 22-019205-008 | B1.1 | Unpublished data |
| 22-019312-001 | B1.1 | Unpublished data |
| 22-019312-002 | B1.1 | Unpublished data |
| 22-019846-002 | B2.1 | Unpublished data |
| 22-020919-001 | B2.1 | Unpublished data |
| 22-020919-002 | B1.1 | Unpublished data |
| 22-021121-001 | A1 | Unpublished data |
| 22-021276-001 | B3.2 | Unpublished data |
| 22-021277-001 | B3.2 | Unpublished data |

| 22-022114-001 | B3.2 | Unpublished data |
| --- | --- | --- |
| 22-023116-001 | B3.2 | Unpublished data |
| 22-024871-001 | A1 | Unpublished data |
| 22-024871-002 | A1 | Unpublished data |
| 22-024871-003 | A1 | Unpublished data |
| 22-024871-004 | A1 | Unpublished data |
| 22-024871-005 | A1 | Unpublished data |
| 22-024871-006 | A1 | Unpublished data |
| 22-024871-007 | A1 | Unpublished data |
| 22-024871-008 | A1 | Unpublished data |
| 22-024871-009 | A1 | Unpublished data |
| 22-024871-010 | A1 | Unpublished data |
| 22-024871-011 | A1 | Unpublished data |
| 22-024871-012 | A1 | Unpublished data |
| 22-024871-013 | A1 | Unpublished data |
| 22-024871-014 | A1 | Unpublished data |
| 22-024871-015 | A1 | Unpublished data |
| 22-024871-016 | A1 | Unpublished data |
| 22-024871-017 | A1 | Unpublished data |
| 22-024871-018 | A1 | Unpublished data |
| 22-024871-019 | A1 | Unpublished data |
| 22-024975-001 | B1.3 | Unpublished data |
| 22-024975-002 | B1.3 | Unpublished data |
| 22-024975-003 | B1.3 | Unpublished data |
| 22-024975-005 | B1.3 | Unpublished data |
| 22-024975-009 | B1.3 | Unpublished data |
| 22-024975-016 | B1.3 | Unpublished data |
| 22-026129-002 | A1 | Unpublished data |
| 22-026130-001 | B1.3 | Unpublished data |
| 22-026731-001 | B3.2 | Unpublished data |
| 22-026731-002 | B3.2 | Unpublished data |
| 22-026969-001 | B3.2 | Unpublished data |
| 22-026969-002 | B3.2 | Unpublished data |
| 22-027222-001 | B1.3 | Unpublished data |
| 22-027249-001 | B1.3 | Unpublished data |
| 22-027250-001 | B1.3 | Unpublished data |
| 22-012000-004 | B2.1 | SRR24843332 |
| 22-012931-001 | B2.1 | SRR24843132 |
| 22-027406-001 | B1.2 | Unpublished data |
| 22-027406-002 | B1.2 | Unpublished data |
| 22-027484-001 | B1.3 | Unpublished data |
| 22-027484-002 | B1.3 | Unpublished data |
| 22-027571-001 | B1.3 | Unpublished data |
| 22-027890-001 | B1.2 | Unpublished data |
| 22-028118-001 | B3.2 | Unpublished data |
| 22-028118-002 | B3.2 | Unpublished data |
| 22-028121-001 | B1.3 | Unpublished data |

| 22-028122-001 | B1.3 | Unpublished data |
| --- | --- | --- |
| 22-028315-001 | B1.3 | Unpublished data |
| 22-028315-002 | B1.3 | Unpublished data |
| 22-028574-001 | B1.3 | Unpublished data |
| 22-028671-001 | B1.3 | Unpublished data |
| 22-028671-002 | B1.3 | Unpublished data |
| 22-028673-002 | B1.3 | Unpublished data |
| 22-028681-001 | B1.3 | Unpublished data |
| 22-028681-002 | B1.3 | Unpublished data |
| 22-028827-008 | B4.1 | Unpublished data |
| 22-028827-009 | B4.1 | Unpublished data |
| 22-028827-010 | B4.1 | Unpublished data |
| 22-028827-011 | B4.1 | Unpublished data |
| 22-028829-002 | B4.1 | Unpublished data |
| 22-028829-004 | B1.3 | Unpublished data |
| 22-028829-005 | B2.2 | Unpublished data |
| 22-029187-001 | B1.3 | Unpublished data |
| 22-029216-001 | B1.3 | Unpublished data |
| 22-029324-002 | B3.2 | Unpublished data |
| 22-029336-002 | B1.3 | Unpublished data |
| 22-029336-004 | B1.3 | Unpublished data |
| 22-029610-001 | B1.3 | Unpublished data |
| 22-029902-001 | B1.3 | Unpublished data |
| 22-029902-002 | B1.3 | Unpublished data |
| 22-030040-001 | B1.3 | Unpublished data |
| 22-030041-001 | B1.3 | Unpublished data |
| 22-030257-001 | B1.3 | Unpublished data |
| 22-030264-001 | A1 | Unpublished data |
| 22-030459-002 | B2.2 | Unpublished data |
| 22-030855-001 | B1.2 | Unpublished data |
| 22-030855-002 | B1.3 | Unpublished data |
| 22-031157-002 | B3.2 | Unpublished data |
| 22-031242-001 | B1.3 | Unpublished data |
| 22-031242-007 | B1.3 | Unpublished data |
| 22-031744-001 | B1.3 | Unpublished data |
| 22-031745-001 | B3.2 | Unpublished data |
| 22-031760-001 | B1.3 | Unpublished data |
| 22-032045-001 | B1.2 | Unpublished data |
| 22-032045-002 | B1.2 | Unpublished data |
| 22-032046-001 | B1.3 | Unpublished data |
| 22-032373-001 | B3.2 | Unpublished data |
| 22-032452-001 | B1.3 | Unpublished data |
| 22-032452-002 | B1.3 | Unpublished data |
| 22-032452-003 | B1.3 | Unpublished data |
| 22-032710-001 | B3.2 | Unpublished data |
| 22-032849-001 | B3.2 | Unpublished data |
| 22-032849-002 | B3.2 | Unpublished data |

| 22-032849-004 | B1.3 | Unpublished data |
| --- | --- | --- |
| 22-032511-008 | B3.2 | Unpublished data |
| 22-032511-020 | B3.2 | Unpublished data |
| 22-032511-067 | B1.3 | Unpublished data |
| 22-032511-070 | B1.3 | Unpublished data |
| 22-032511-071 | B1.3 | Unpublished data |
| 22-032511-077 | B1.3 | Unpublished data |
| 22-032511-097 | B1.3 | Unpublished data |
| 22-032511-101 | B3.2 | Unpublished data |
| 22-032511-114 | B1.3 | Unpublished data |
| 22-032511-165 | B1.3 | Unpublished data |
| 22-032511-174 | B1.3 | Unpublished data |
| 22-032511-190 | B1.3 | Unpublished data |
| 22-032745-012 | B1.3 | Unpublished data |
| 22-032745-018 | B1.2 | Unpublished data |
| 22-032745-034 | B1.3 | Unpublished data |
| 22-032745-038 | B3.2 | Unpublished data |
| 22-032745-046 | B1.3 | Unpublished data |
| 22-032745-055 | B1.3 | Unpublished data |
| 22-033009-010 | B3.3 | Unpublished data |
| 22-033009-014 | B1.3 | Unpublished data |
| 22-033080-001 | B1.3 | Unpublished data |
| 22-033728-010 | B1.3 | Unpublished data |
| 22-033812-001 | B1.3 | Unpublished data |
| 22-034080-011 | B3.2 | Unpublished data |
| 22-034080-032 | B3.2 | Unpublished data |
| 22-034080-051 | B1.3 | Unpublished data |
| 22-034926-001 | B2.2 | Unpublished data |
| 22-035164-002 | B1.1 | Unpublished data |
| 22-035164-003 | B1.1 | Unpublished data |
| 22-035164-004 | B1.1 | Unpublished data |
| 22-035164-007 | B1.1 | Unpublished data |
| 22-035164-008 | B1.1 | Unpublished data |
| 22-035164-009 | B1.1 | Unpublished data |
| 22-035164-010 | B1.1 | Unpublished data |
| 22-035164-011 | B1.1 | Unpublished data |
| 22-035164-012 | B1.1 | Unpublished data |
| 22-035164-013 | B1.1 | Unpublished data |
| 22-035164-014 | B1.1 | Unpublished data |
| 22-035404-002 | B3.3 | Unpublished data |
| 22-035442-001 | Neo | Unpublished data |
| 22-035443-007 | Neo | Unpublished data |
| 22-035505-001 | B1.3 | Unpublished data |
| 22-035505-002 | B3.2 | Unpublished data |
| 22-035724-001 | B1.3 | Unpublished data |
| 22-035727-001 | B1.3 | Unpublished data |
| 22-035805-002 | B1.3 | Unpublished data |

| 22-035805-003 | B1.3 | Unpublished data |
| --- | --- | --- |
| 22-035805-004 | B1.3 | Unpublished data |
| 22-028755-003 | B2.2 | Unpublished data |
| 22-034674-001 | B1.3 | Unpublished data |
| 22-036371-001 | B1.1 | Unpublished data |
| 22-036371-002 | B1.1 | Unpublished data |
| 22-036762-002 | B1.3 | Unpublished data |
| 22-036798-001 | B3.2 | Unpublished data |
| 22-037161-001 | B1.1 | Unpublished data |
| 22-037236-004 | B3.2 | Unpublished data |
| 22-037240-001 | B2.2 | Unpublished data |
| 22-037240-002 | B3.2 | Unpublished data |
| 22-037240-003 | B3.2 | Unpublished data |
| 22-037438-001 | B3.2 | Unpublished data |
| 22-037439-001 | B3.2 | Unpublished data |
| 22-037698-001 | B1.1 | Unpublished data |
| 22-036680-001 | B1.3 | Unpublished data |
| 22-037787-001 | B3.2 | Unpublished data |
| 22-037797-001 | B1.3 | Unpublished data |
| 22-037799-001 | B2.2 | Unpublished data |
| 22-037822-001 | Minor32 | Unpublished data |
| 22-038202-001 | B3.2 | Unpublished data |
| 22-038267-001 | Minor28 | Unpublished data |
| 22-038368-001 | B2.2 | Unpublished data |
| 22-038370-001 | B1.2 | Unpublished data |
| 22-038433-002 | Neo | Unpublished data |
| 22-038557-001 | B2.2 | Unpublished data |
| 22-038557-002 | B3.2 | Unpublished data |
| 22-038557-003 | minor19 | Unpublished data |
| 22-038557-004 | B2.2 | Unpublished data |
| 22-038564-001 | B3.2 | Unpublished data |
| 22-038564-002 | B3.2 | Unpublished data |
| 22-038564-003 | B2.2 | Unpublished data |
| 22-038564-004 | B2.2 | Unpublished data |
| 22-038564-005 | B2.2 | Unpublished data |
| 22-039758-001 | B2.2 | Unpublished data |
| 22-039758-001 | B2.2 | Unpublished data |
| 22-039759-003 | B2.2 | Unpublished data |
| 22-039759-003 | B2.2 | Unpublished data |
| 22-039760-001 | B3.2 | Unpublished data |
| 22-039793-002 | Minor30 | Unpublished data |
| 22-039817-001 | B3.2 | Unpublished data |
| 22-039817-001 | B3.2 | Unpublished data |
| 22-039347-001 | B1.1 | Unpublished data |
| 22-039347-002 | B1.1 | Unpublished data |
| 22-039347-003 | B1.1 | Unpublished data |
| 22-039352-001 | B1.3 | Unpublished data |

| 22-039352-002 | B1.3 | Unpublished data |
| --- | --- | --- |
| 22-039352-003 | B1.3 | Unpublished data |
| 22-039352-004 | B1.3 | Unpublished data |
| 22-039352-005 | B1.3 | Unpublished data |
| 22-039352-006 | B1.3 | Unpublished data |
| 22-039352-007 | B1.3 | Unpublished data |
| 22-039352-008 | B1.3 | Unpublished data |
| 22-039352-009 | B1.3 | Unpublished data |
| 22-039352-011 | B1.3 | Unpublished data |
| 22-039352-013 | B1.3 | Unpublished data |
| 22-039352-014 | B1.3 | Unpublished data |
| 22-039352-015 | B1.3 | Unpublished data |
| 22-039352-016 | B1.3 | Unpublished data |
| 22-039352-017 | B1.3 | Unpublished data |
| 22-039352-018 | B1.3 | Unpublished data |
| 22-039352-019 | B1.3 | Unpublished data |
| 22-039352-020 | B1.3 | Unpublished data |
| 22-039392-002 | B2.2 | Unpublished data |
| 22-038820-003 | B2.2 | Unpublished data |
| 22-038820-008 | B2.2 | Unpublished data |
| 22-038821-004 | B3.2 | Unpublished data |
| 22-038821-005 | B2.2 | Unpublished data |
| 22-038821-007 | B3.4 | Unpublished data |
| 22-038821-008 | B3.2 | Unpublished data |
| 22-038821-010 | B3.2 | Unpublished data |
| 22-038821-011 | B3.2 | Unpublished data |
| 22-038821-012 | B3.2 | Unpublished data |
| 22-038821-013 | B3.2 | Unpublished data |
| 22-038821-016 | B2.2 | Unpublished data |
| 22-038821-019 | B3.2 | Unpublished data |
| 22-038821-020 | B3.2 | Unpublished data |
| 22-038821-021 | B2.2 | Unpublished data |
| 22-038821-022 | B2.2 | Unpublished data |
| 22-038821-023 | B3.2 | Unpublished data |
| 22-038821-024 | B3.2 | Unpublished data |
| 22-038821-025 | B3.2 | Unpublished data |
| 22-038821-026 | B3.2 | Unpublished data |
| 22-038821-028 | B3.4 | Unpublished data |
| 22-038821-029 | B2.2 | Unpublished data |
| 22-038821-030 | B3.2 | Unpublished data |
| 22-038821-031 | B3.2 | Unpublished data |
| 22-038821-032 | B2.2 | Unpublished data |
| 22-038821-034 | B2.2 | Unpublished data |
| 22-038821-038 | B2.2 | Unpublished data |
| 22-038821-039 | B3.2 | Unpublished data |
| 22-038821-042 | B3.2 | Unpublished data |
| 22-038821-044 | B3.2 | Unpublished data |

| 22-038821-046 | B3.2 | Unpublished data |
| --- | --- | --- |
| 22-038821-048 | B2.2 | Unpublished data |
| 22-038821-049 | B2.2 | Unpublished data |
| 22-038821-050 | B3.2 | Unpublished data |
| 22-038821-051 | B3.2 | Unpublished data |
| 22-038821-052 | B2.2 | Unpublished data |
| 22-038821-053 | B2.2 | Unpublished data |
| 22-038821-054 | B3.2 | Unpublished data |
| 22-038821-055 | B2.2 | Unpublished data |
| 22-038821-056 | B2.2 | Unpublished data |
| 22-038821-058 | B3.6 | Unpublished data |
| 22-038827-002 | B3.2 | Unpublished data |
| 22-038827-003 | B3.2 | Unpublished data |
| 22-038827-008 | B3.2 | Unpublished data |
| 22-038827-009 | B2.2 | Unpublished data |
| 22-038827-011 | B3.5 | Unpublished data |
| 22-039197-002 | B1.1 | Unpublished data |
| 22-039689-001 | B1.1 | Unpublished data |
| 22-039689-002 | B1.1 | Unpublished data |
| 22-039689-003 | B1.1 | Unpublished data |
| 22-039693-001 | B1.3 | Unpublished data |
| 22-040349-001 | B1.1 | Unpublished data |
| 22-040462-002 | B2.2 | Unpublished data |
| 22-040520-001 | B3.2 | Unpublished data |
| 22-040522-003 | B3.4 | Unpublished data |
| 22-029336-017 | B1.3 | Unpublished data |
| 22-029724-002 | B1.3 | Unpublished data |
| 22-029725-007 | B3.2 | Unpublished data |
| 22-029725-008 | B3.2 | Unpublished data |
| 22-031456-001 | B1.3 | Unpublished data |
| 22-039178-001 | B3.2 | Unpublished data |
| 22-039178-002 | B3.2 | Unpublished data |
| 22-040571-002 | Minor25 | Unpublished data |
| 22-040685-001 | B1.3 | Unpublished data |
| 22-040701-002 | B1.3 | Unpublished data |
| 22-040776-001 | B1.1 | Unpublished data |
| 22-040782-001 | B1.3 | Unpublished data |
| 22-040785-001 | B3.2 | Unpublished data |
| 22-040785-002 | B3.2 | Unpublished data |
| 22-040786-001 | B3.2 | Unpublished data |
| 22-040790-001 | B3.2 | Unpublished data |
| 22-040790-003 | B3.2 | Unpublished data |
| 22-040792-001 | B2.2 | Unpublished data |
| 22-040793-003 | B2.2 | Unpublished data |
| 22-040793-004 | B3.2 | Unpublished data |
| 22-040793-005 | B3.2 | Unpublished data |
| 22-040806-001 | B3.2 | Unpublished data |

| 22-040814-001 | B3.2 | Unpublished data |
| --- | --- | --- |
| 22-040816-001 | B3.2 | Unpublished data |
| 22-040817-014 | B1.3 | Unpublished data |
| 22-040817-016 | B1.3 | Unpublished data |
| 22-040946-001 | B2.2 | Unpublished data |
| 22-040817-006 | B1.3 | Unpublished data |
| 22-040817-007 | B1.3 | Unpublished data |
| 22-040817-009 | B1.3 | Unpublished data |
| 22-040817-015 | B1.3 | Unpublished data |
| 22-040820-002 | B1.3 | Unpublished data |
| 22-041054-001 | B1.1 | Unpublished data |
| 22-041054-002 | B1.1 | Unpublished data |
| 22-041054-003 | B1.1 | Unpublished data |
| 22-041054-004 | B1.1 | Unpublished data |
| 22-041054-005 | B1.1 | Unpublished data |
| 22-041056-001 | B1.1 | Unpublished data |
| 22-041057-001 | B3.2 | Unpublished data |
| 22-041059-001 | B3.2 | Unpublished data |
| 22-041306-001 | B3.2 | Unpublished data |
| 22-041306-002 | B3.6 | Unpublished data |
| 22-041373-003 | B2.2 | Unpublished data |
| 22-041777-001 | B3.4 | Unpublished data |
| 22-041884-001 | B3.2 | Unpublished data |
| 22-041996-001 | B1.1 | Unpublished data |
| 22-041760-007 | B3.2 | Unpublished data |
| 22-041761-001 | B2.2 | Unpublished data |
| 22-041761-007 | B3.2 | Unpublished data |
| 22-041761-016 | B2.2 | Unpublished data |
| 22-041762-001 | B3.2 | Unpublished data |
| 22-041762-002 | B2.2 | Unpublished data |
| 22-041762-003 | B2.2 | Unpublished data |
| 22-041762-004 | B3.2 | Unpublished data |
| 22-041762-005 | B3.2 | Unpublished data |
| 22-041762-006 | B3.2 | Unpublished data |
| 22-041764-001 | B3.2 | Unpublished data |
| 22-042176-001 | B3.4 | Unpublished data |
| 22-042176-002 | B3.4 | Unpublished data |
| 23-000235-001 | B3.2 | Unpublished data |
| 23-000269-001 | A3 | Unpublished data |
| 23-000335-001 | B1.3 | Unpublished data |
| 23-000394-001 | B3.4 | Unpublished data |
| 23-000627-001 | B3.2 | Unpublished data |
| 23-000627-002 | B3.2 | Unpublished data |
| 23-000627-003 | B3.2 | Unpublished data |
| 23-000627-004 | B3.2 | Unpublished data |
| 23-000627-005 | B3.2 | Unpublished data |
| 23-000627-006 | B3.2 | Unpublished data |

| 23-000627-007 | B3.2 | Unpublished data |
| --- | --- | --- |
| 23-000794-001 | B1.1 | Unpublished data |
| 22-030404-001 | B1.3 | Unpublished data |
| 22-031157-003 | B3.2 | Unpublished data |
| 22-031242-002 | B1.2 | Unpublished data |
| 22-031242-003 | B1.1 | Unpublished data |
| 22-031242-004 | B1.3 | Unpublished data |
| 22-031242-005 | B1.3 | Unpublished data |
| 23-001508-001 | B3.2 | Unpublished data |
| 23-001510-001 | B3.4 | Unpublished data |
| 23-001511-001 | B2.2 | Unpublished data |
| 23-001512-001 | B3.2 | Unpublished data |
| 23-001513-001 | B3.2 | Unpublished data |
| 23-001514-001 | B3.2 | Unpublished data |
| 23-001516-001 | B3.4 | Unpublished data |
| 23-001518-001 | B3.2 | Unpublished data |
| 23-001520-001 | B3.2 | Unpublished data |
| 23-001521-001 | B3.4 | Unpublished data |
| 23-001859-001 | B3.2 | Unpublished data |
| 23-002039-001 | B3.2 | Unpublished data |
| 23-002039-002 | B3.2 | Unpublished data |
| 22-039392-001 | B3.2 | Unpublished data |
| 22-039392-003 | B3.2 | Unpublished data |
| 23-002620-001 | B1.3 | Unpublished data |
| 23-002620-002 | B1.3 | Unpublished data |
| 23-002620-003 | B1.1 | Unpublished data |
| 23-002620-004 | B1.3 | Unpublished data |
| 23-002620-005 | B1.3 | Unpublished data |
| 23-002620-006 | B1.3 | Unpublished data |
| 23-002620-007 | B1.1 | Unpublished data |
| 23-002620-008 | B1.3 | Unpublished data |
| 23-002620-009 | B1.3 | Unpublished data |
| 23-002620-010 | B1.3 | Unpublished data |
| 23-002621-001 | B1.3 | Unpublished data |
| 23-002621-002 | B1.3 | Unpublished data |
| 23-002621-004 | B1.3 | Unpublished data |
| 23-002621-005 | B1.3 | Unpublished data |
| 23-002621-006 | B1.3 | Unpublished data |
| 23-002621-007 | B1.3 | Unpublished data |
| 23-002621-008 | B1.3 | Unpublished data |
| 23-002621-010 | B1.3 | Unpublished data |
| 23-002623-001 | B1.3 | Unpublished data |
| 23-002624-001 | B1.3 | Unpublished data |
| 23-002625-001 | B1.3 | Unpublished data |
| 23-002662-001 | B3.2 | Unpublished data |
| 23-002662-002 | B3.2 | Unpublished data |
| 23-002690-001 | B3.2 | Unpublished data |

| 23-002711-001 | B3.4 | Unpublished data |
| --- | --- | --- |
| 22-032511-043 | B1.3 | Unpublished data |
| 22-032511-051 | B1.3 | Unpublished data |
| 22-032511-063 | B3.2 | Unpublished data |
| 22-032511-098 | B3.2 | Unpublished data |
| 22-032511-101 | B3.2 | Unpublished data |
| 22-032511-149 | B1.3 | Unpublished data |
| 22-032511-168 | B1.3 | Unpublished data |
| 22-032511-170 | B1.3 | Unpublished data |
| 22-032745-002 | B3.2 | Unpublished data |
| 23-001343-001 | B1.3 | Unpublished data |
| 23-001343-002 | B1.3 | Unpublished data |
| 23-001343-003 | B1.3 | Unpublished data |
| 23-001343-004 | B1.3 | Unpublished data |
| 23-001343-005 | B1.3 | Unpublished data |
| 23-001343-006 | B1.3 | Unpublished data |
| 23-001343-007 | B1.3 | Unpublished data |
| 23-001343-008 | B1.3 | Unpublished data |
| 23-001343-009 | B1.3 | Unpublished data |
| 23-001343-010 | B1.3 | Unpublished data |
| 23-001343-011 | B1.3 | Unpublished data |
| 23-001343-012 | B1.3 | Unpublished data |
| 23-001343-013 | B1.3 | Unpublished data |
| 23-001343-014 | B1.3 | Unpublished data |
| 23-001343-015 | B1.3 | Unpublished data |
| 23-001343-016 | B1.3 | Unpublished data |
| 23-001343-017 | B1.3 | Unpublished data |
| 23-001343-019 | B1.3 | Unpublished data |
| 23-001343-020 | B1.3 | Unpublished data |
| 23-002626-001 | B3.2 | Unpublished data |
| 23-003026-001 | B3.2 | Unpublished data |
| 23-003091-002 | B1.3 | Unpublished data |
| 23-003553-001 | B3.2 | Unpublished data |
| 23-003620-001 | B1.3 | Unpublished data |
| 23-003620-002 | B1.3 | Unpublished data |
| 23-003815-001 | B3.4 | Unpublished data |
| 22-034303-001 | B2.2 | Unpublished data |
| 22-034304-005 | B3.2 | Unpublished data |
| 23-006279-001 | B3.2 | Unpublished data |
| 23-006381-001 | B1.1 | Unpublished data |
| 23-006381-002 | B1.1 | Unpublished data |
| 23-006381-003 | B1.1 | Unpublished data |
| 23-006381-004 | B1.1 | Unpublished data |
| 23-006381-005 | B1.1 | Unpublished data |
| 22-034829-003 | B3.2 | Unpublished data |
| 22-034829-007 | B3.2 | Unpublished data |
| 22-034928-001 | B3.2 | Unpublished data |

| 23-007195-001 | B3.5 | EPI2613854 |
| --- | --- | --- |
| 23-007195-003 | B3.5 | EPI2613862 |
| 23-007195-005 | B3.5 | EPI2613870 |
| 23-007195-008 | B3.5 | EPI2613878 |
| 23-006381-003 | B1.1 | Unpublished data |
| 23-006381-004 | B1.1 | Unpublished data |
| 23-007350-001 | B3.4 | EPI2613886 |
| 23-007375-001 | B1.3 | EPI2613894 |
| 23-007468-001 | B3.2 | EPI2613902 |
| 23-007781-002 | B3.2 | EPI2613926 |
| 22-035805-001 | B1.3 | Unpublished data |
| 22-035805-003 | B1.3 | Unpublished data |
| 22-035805-004 | B1.3 | Unpublished data |
| 22-035805-005 | B1.3 | Unpublished data |
| 22-037240-002 | B3.2 | Unpublished data |
| 23-008405-001 | B1.3 | Unpublished data |
| 23-008517-001 | B3.6 | EPI2614022 |
| 22-038820-002 | B3.2 | Unpublished data |
| 22-038820-004 | B2.2 | Unpublished data |
| 22-038820-006 | B3.2 | Unpublished data |
| 22-038820-010 | B2.2 | Unpublished data |
| 22-038827-001 | B3.2 | Unpublished data |
| 22-038827-002 | B3.2 | Unpublished data |
| 22-038827-003 | B3.2 | Unpublished data |
| 22-038827-004 | B2.2 | Unpublished data |
| 22-038827-005 | B3.2 | Unpublished data |
| 22-038827-006 | Neo | Unpublished data |
| 22-038827-007 | B3.2 | Unpublished data |
| 22-038827-008 | B3.2 | Unpublished data |
| 22-038827-009 | B2.2 | Unpublished data |
| 22-038827-010 | B2.2 | Unpublished data |
| 22-038828-001 | B3.2 | Unpublished data |
| 22-040427-001 | B3.2 | Unpublished data |
| 22-040427-002 | B3.2 | Unpublished data |
| 22-040427-003 | B2.2 | Unpublished data |
| 22-038821-002 | B2.2 | Unpublished data |
| 22-038821-003 | B3.6 | Unpublished data |
| 22-038821-006 | B3.2 | Unpublished data |
| 22-038821-009 | B3.2 | Unpublished data |
| 22-038821-014 | B3.2 | Unpublished data |
| 22-038821-033 | B3.2 | Unpublished data |
| 22-038821-036 | B2.2 | Unpublished data |
| 22-038821-041 | B2.2 | Unpublished data |
| 22-038821-047 | B3.2 | Unpublished data |
| 22-038821-054 | B3.2 | Unpublished data |
| 22-038821-057 | B3.2 | Unpublished data |
| 22-038821-059 | Minor28 | Unpublished data |

| 22-039225-001 | B3.4 | Unpublished data |
| --- | --- | --- |
| 22-039348-001 | B3.2 | Unpublished data |
| 22-039349-001 | B3.2 | Unpublished data |
| 22-039351-001 | B3.2 | Unpublished data |
| 22-039351-003 | B3.2 | Unpublished data |
| 22-039351-004 | B2.2 | Unpublished data |
| 22-039351-005 | B3.6 | Unpublished data |
| 22-039354-001 | B3.6 | Unpublished data |
| 22-039357-005 | B3.2 | Unpublished data |
| 23-010018-001 | B3.2 | EPI2614134 |
| 23-010337-001 | Minor28 | Unpublished data |
| 23-010384-001 | B3.2 | Unpublished data |
| 22-039743-001 | B3.2 | Unpublished data |
| 22-039754-001 | B3.2 | Unpublished data |
| 22-039758-002 | B2.2 | Unpublished data |
| 22-039759-001 | B3.2 | Unpublished data |
| 22-039760-002 | B2.2 | Unpublished data |
| 22-039793-002 | B3.2 | Unpublished data |
| 23-010338-001 | B1.3 | Unpublished data |
| 23-010832-001 | B3.2 | EPI2614158 |
| 23-010832-002 | B3.2 | EPI2614166 |
| 22-039351-003 | B3.2 | Unpublished data |
| 22-039351-004 | B2.2 | Unpublished data |
| 22-039351-005 | B3.6 | Unpublished data |
| 22-039353-001 | B2.2 | Unpublished data |
| 22-040349-001 | B1.1 | Unpublished data |
| 22-040522-004 | minor19 | Unpublished data |
| 22-040523-001 | B3.2 | Unpublished data |
| 23-000269-001 | A3 | Unpublished data |
| 23-011213-001 | B3.2 | EPI2614206 |
| 23-011253-001 | B1.1 | Unpublished data |
| 22-039760-002 | B2.2 | Unpublished data |
| 22-040817-002 | B1.3 | Unpublished data |
| 22-040817-005 | B1.3 | Unpublished data |
| 22-040817-011 | B1.3 | Unpublished data |
| 22-040817-016 | B1.3 | Unpublished data |
| 23-011253-001 | B1.1 | Unpublished data |
| 23-011257-001 | B3.2 | Unpublished data |
| 23-013080-001 | B2.2 | Unpublished data |
| 23-013080-002 | B3.2 | Unpublished data |
| 23-013080-003 | B3.2 | Unpublished data |
| 23-013080-004 | B3.2 | Unpublished data |
| 23-013080-005 | B3.2 | Unpublished data |
| 23-013080-006 | B3.2 | Unpublished data |
| 23-013080-007 | B3.2 | Unpublished data |
| 23-013080-008 | B1.3 | Unpublished data |
| 23-013080-009 | B3.2 | Unpublished data |

| 23-013080-010 | B3.2 | Unpublished data |
| --- | --- | --- |
| 23-013080-011 | B3.2 | Unpublished data |
| 22-040347-001 | B2.2 | Unpublished data |
| 22-040817-001 | B1.3 | Unpublished data |
| 22-040817-002 | B1.3 | Unpublished data |
| 22-040817-004 | B1.3 | Unpublished data |
| 22-040817-006 | B1.3 | Unpublished data |
| 22-040817-007 | B1.3 | Unpublished data |
| 22-040817-010 | B1.3 | Unpublished data |
| 22-040817-011 | B1.3 | Unpublished data |
| 22-040817-012 | B1.3 | Unpublished data |
| 22-041765-001 | B3.2 | Unpublished data |
| 23-001343-018 | B3.5 | Unpublished data |
| 23-002056-001 | B1.3 | Unpublished data |
| 23-002620-007 | B1.1 | Unpublished data |
| 23-002662-001 | B3.2 | Unpublished data |
| 23-004671-001 | B2.1 | Unpublished data |
| 23-004671-001 | B2.1 | Unpublished data |
| 23-004671-001 | B2.1 | Unpublished data |
| 23-011253-001 | B1.1 | Unpublished data |
| 23-011253-003 | B1.2 | Unpublished data |
| 22-040817-011 | B1.3 | Unpublished data |
| 23-001343-016 | B1.3 | Unpublished data |
| 23-001845-002 | B3.2 | Unpublished data |
| 23-002620-007 | B1.1 | Unpublished data |
| 23-002621-009 | B1.3 | Unpublished data |
| 23-002662-001 | B3.2 | Unpublished data |
| 23-015429-001 | minor36 | EPI2614566 |
| 23-016157-001 | B3.2 | Unpublished data |
| 23-016157-005 | B2.2 | Unpublished data |
| 23-016157-006 | B3.2 | Unpublished data |
| 23-016157-009 | B2.2 | Unpublished data |
| 23-016157-012 | B3.2 | Unpublished data |
| 23-016159-001 | B1.3 | Unpublished data |
| 23-016159-002 | B1.3 | Unpublished data |
| 23-016159-003 | B1.3 | Unpublished data |
| 23-016159-004 | B1.3 | Unpublished data |
| 23-016159-005 | B1.3 | Unpublished data |
| 23-016159-006 | B1.3 | Unpublished data |
| 23-016159-007 | minor24 | Unpublished data |
| 23-016159-008 | B1.3 | Unpublished data |
| 23-016159-009 | B1.3 | Unpublished data |
| 23-016159-010 | minor24 | Unpublished data |
| 23-016159-011 | B1.3 | Unpublished data |
| 23-016159-012 | B1.3 | Unpublished data |
| 23-016159-013 | B1.3 | Unpublished data |
